# Validation of 4 Risk Stratification Tools for Delirium in the Emergency Department

**DOI:** 10.1001/jamanetworkopen.2025.40920

**Published:** 2025-11-03

**Authors:** Manuela Bartolacci, Kayla P. Carpenter, Molly M. Jeffery, Aidan F. Mullan, Christopher R. Carpenter, Fernanda Bellolio

**Affiliations:** 1 Department of Emergency Medicine, Mayo Clinic, Rochester, Minnesota; 2 Department of Internal Medicine and Medical Therapy, University of Pavia, Pavia, Italy; 3 Department of Industrial and Information Engineering, University of Pavia, Pavia, Italy; 4 Robert D. and Patricia E. Kern Center for the Science of Health Care Delivery, Mayo Clinic, Rochester, Minnesota; 5 Department of Quantitative Health Sciences, Mayo Clinic, Rochester, Minnesota; 6 Robert and Arlene Kogod Center on Aging, Mayo Clinic, Rochester, Minnesota

## Abstract

**Importance:**

Validation of prognostic tools is an essential component of their integration into clinical practice.

**Objective:**

To validate 4 delirium risk stratification tools in an independent emergency department (ED) cohort.

**Design, Setting, and Participants:**

This prognostic study included a retrospective cohort of patients 65 years or older who presented to an academic ED between January 1, 2021, and December 31, 2024. Data were analyzed from February 10 to August 21, 2025.

**Exposure:**

Delirium screening included the Delirium Triage Screen followed by the brief Confusional Assessment Method as part of the standard of care. Four delirium risk scores were obtained, including the Kennedy rule, the Zucchelli rule, the Mayo Delirium Prediction (MDP) tools, and the Recognizing Delirium in Emergency Medicine (REDEEM) score.

**Main Outcomes and Measures:**

Main outcomes included risk score performances and diagnostic test accuracy measured using the area under the receiver operating characteristics curve (AUROC), sensitivity, specificity, negative (NPV) and positive (PPV) predictive values, and positive and negative likelihood ratios (LRs) with 95% CIs. Secondary outcomes were discriminative and calibration measures.

**Results:**

A total of 44 578 patients were included, of whom 1701 (3.8%) were diagnosed with delirium. The median age was 80.0 (IQR, 75.0-85.0) years, and 22 786 patients (51.1%) were female. The Kennedy rule had an AUROC of 0.777 (95% CI, 0.766-0.789). At a cutoff of 5, sensitivity was 0.55 (95% CI, 0.52-0.57); specificity, 0.85 (95% CI, 0.84-0.85); positive LR, 3.54 (95% CI, 3.37-3.72); negative LR, 0.54 (95% CI, 0.51-0.57); PPV, 0.12 (95% CI, 0.12-0.13); and NPV, 0.98 (95% CI, 0.98-0.98). The Zucchelli tool had an AUROC of 0.701 (95% CI, 0.686-0.713). At a cutoff of 5, sensitivity was 0.68 (95% CI, 0.66-0.70); specificity, 0.66 (95% CI, 0.65-0.66); positive LR, 1.99 (95% CI, 1.92-2.06); negative LR, 0.49 (95% CI, 0.45-0.52); PPV, 0.07 (95% CI, 0.07-0.08); and NPV, 0.98 (95% CI, 0.98-0.98). The MDP tool had an AUROC of 0.898 (95% CI, 0.891-0.905). At a 30% cutoff, sensitivity was 0.51 (95% CI, 0.48-0.53); specificity, 0.95 (95% CI, 0.95-0.95); positive LR, 9.69 (95% CI, 9.11-10.31); negative LR, 0.52 (95% CI, 0.50-0.55); PPV, 0.28 (95% CI, 0.26-0.29); and NPV, 0.98 (95% CI, 0.98-0.98). The REDEEM score demonstrated the highest AUROC at 0.921 (95% CI, 0.914-0.929). Using a cutoff of 11 or greater, sensitivity was 0.83 (95% CI, 0.81-0.85); specificity, 0.92 (95% CI, 0.91-0.92), negative LR, 0.18 (95% CI, 0.17-0.20); positive LR, 9.91 (95% CI, 9.54-10.29); PPV, 0.28 (95% CI, 0.27-0.29); and NPV, 0.99 (95% CI, 0.99-0.99).

**Conclusions and Relevance:**

In this prognostic study comparing 4 delirium tools to improve delirium detection and identify high-risk patients, the REDEEM score and the MDP tool had better performance for delirium detection in the ED. These findings lay the groundwork for integrating validated risk stratification into ED workflows to improve early delirium detection and inform prevention strategies for high-risk older adults.

## Key Points

**Question:**

Are previously developed delirium risk stratification tools applicable to a different setting in the emergency department (ED)?

**Findings:**

In this prognostic study including 44 578 patients, 3 of the 4 delirium scores demonstrated performance that was comparable with the scores attained in the original studies. The Recognizing Delirium in Emergency Medicine (REDEEM) score demonstrated the strongest predictive capacity, and the Mayo Delirium Prediction (MDP) tool, despite being developed for hospitalized patients, exhibited a high degree of accuracy in detecting delirium in ED patients.

**Meaning:**

These findings suggest that the REDEEM score and the MDP tool may be valid instruments to potentially improve delirium detection rates in the ED.

## Teaser

This cohort study validates 4 different risk stratification tools for detecting delirium among patients in the emergency department.

## Introduction

Delirium is an acute and fluctuating disturbance of attention, awareness, and cognition typically resulting from underlying medical conditions or drug effects. It is a clinical diagnosis made exclusively at the bedside and manifests in 2 primary motor subtypes: hyperactive and hypoactive, the latter being more common and often missed.^1,2^

The prevalence of delirium is approximately 10% to 15% in geriatric emergency departments (EDs).^3,4^ However, delirium is often underrecognized in the ED, going undetected in as many as 83% of cases.^1,5^ Underdiagnosis of ED delirium is multifactorial and includes time and resource constraints, competing priorities, failure to appreciate the consequences of unrecognized delirium, language barriers, inconsistent access to care partners, therapeutic nihilism, and the predominance of hypoactive presentations.^1,6,7,8,9,10,11^

Prompt identification of delirium is crucial, as missed delirium is linked to prolonged hospitalization, cognitive impairment, increased health care costs, decreased long-term functioning, and increased short- and long-term mortality, especially following prolonged episodes.^1,11^ ED delirium is associated with higher 7- and 30-day mortality as well as a 3-fold increase in 6-month mortality among patients discharged from the ED with unrecognized delirium.^1,12,13^

Kennedy et al^14^ have advocated for systematic, evidence-based approaches to delirium detection and prevention, emphasizing that reliable screening is essential to any delirium quality improvement effort. Despite delirium screening being incorporated as a requirement for geriatric EDs, its implementation is challenging and resource consuming, requiring time and dedicated staff.^3,4,15,16,17,18,19^ To compound these challenges, EDs worldwide are overcrowded, and the geriatric population is growing rapidly.^10,17,20^ Identifying patients at high risk for delirium with risk stratification tools could enhance delirium detection and improve the feasibility of screening implementation.^21^ However, developing such a prognostic system is challenging due to the fluctuating nature of delirium.^21^

A recent systematic review^22^ identified 6 delirium risk stratification tools and showed good discriminative performance (area under the receiver operating characteristics curve [AUROC], 0.77-0.90); however, the effects of these on care processes and clinical outcomes remain unproven. Furthermore, calibration of these instruments remains underreported, reflecting a broader issue in tool development and validation where methodology is often poorly reported.^22,23,24,25,26^

Incorporating automated prediction models into electronic health records (EHR) could support real-time risk assessment. For example, the Mayo Delirium Prediction (MDP) tool demonstrate strong predictive performance (AUROC, approximately 0.80) in hospitalized patients.^4,27,28^ However, before integrating such models into clinical practice, their robustness and generalizability must be validated in external cohorts.^22,29^

The objective of this study was to validate 4 different delirium risk stratification tools in an ED cohort different from the original studies cohorts. For this purpose, we selected tools containing variables available at the time of the ED visit that could be implemented in everyday ED practice.^22^ We also included the MDP tool due to its automated nature and its potential applicability both to inpatient and ED settings.

## Methods

This prognostic study adhered to the Transparent Reporting of a Multivariable Prediction Model for Individual Prognosis or Diagnosis (TRIPOD) reporting guideline.^30^ The study was approved by the Institutional Review Board of the Mayo Clinic, Rochester, Minnesota, and all patients included gave authorization for medical record review.

### Delirium Measurement

The cohort used for validation included all consecutive ED encounters of adults 75 years and older who presented to a single academic ED in Minnesota from January 1, 2021, to December 31, 2023, and all consecutive adults 65 years and older from January 1 to December 31, 2024. The primary outcome was delirium status measured with a 2-step delirium screen: the Delirium Triage Screening (DTS) and brief Confusion Assessment Method (bCAM).^3,31,32^ These screenings were conducted by bedside nurses during their clinical shifts. DTS was administered first: a negative result ruled out delirium due to its high sensitivity. If the DTS finding was positive, bCAM was administered; a positive bCAM finding indicated delirium, given its high specificity. Patients with a positive DTS finding but a negative bCAM finding, or a negative bCAM finding without a positive DTS finding, were classified as not having delirium. Patients with multiple screenings during an ED evaluation were classified as being positive for delirium if any result was positive. Patients with no or incomplete DTS and/or bCAM screening were excluded from the primary outcome analysis (16 254 [26.7%] of 60 832 eligible visits). Bedside assessments were regularly performed in 80% of the patients. Those with acute stroke, confusion, speech impairment, and severe trauma were usually not assessed.

### Delirium Stratification Tools Selection

Our primary aim was to validate delirium stratification tools that are applicable to the ED. We selected the following 4 scores summarized in Table 1:

**Table 1.** Characteristics of Delirium Risk Stratification Tools Selected for Validation

Score	Features	Risk categories	Reported performance
Kennedy delirium risk prediction rule	Age 65-69 y (+0 points), 70-79 y (+1 point), 80-89 y (+2 points), and ≥90 y (+3 points); history of dementia (+3 points); history of ischemic stroke or TIA (+2 points); respiratory rate >20 breaths/min (+2 points); suspected infection (+2 points); ED diagnosis of ICH (+5 points)	Low, ≤2 points; moderate, 3-4 points; high, ≥5 points	C statistic, 0.77 (95% CI, 0.71-0.83); Hosmer-Lemeshow test, *P* = .34; cutoff 2, sensitivity = 76.2% and specificity = 65.4%; cutoff 5, sensitivity = 47.6% and specificity = 87.9%
Zucchelli risk prediction tool	Age ≥75 y (+2 points); dementia (+3 points); hearing impairment (+3 points); chronic use of psychotropic drugs (+1 point)	High: ≥3 points for greater sensitivity or ≥5 points for greater specificity	AUROC, 0.893; cutoff 3, sensitivity = 91.9% and specificity = 74.3%; cutoff 5, sensitivity = 73.0% and specificity = 91.4%
REDEEM score	Arrival via EMS (+ 1 point); triage chief complaint of altered mental status (+18 points); ESI level ≥3 (−3 points); oxygen saturation <92% (+2 points); systolic blood pressure <111 mm Hg (+2 points); diastolic blood pressure >99 mm Hg (+1 point); respiratory rate <16 breaths/min (+3 points); respiratory rate >24 breaths/min (+6 points); confusion or disorientation identified during fall risk assessment (+25 points); altered elimination identified during fall risk assessment (+8 points); history of seizures disorders (+4 points)	High, ≥5 points for greater sensitivity or ≥11 points for greater specificity	AUROC, 0.901 (95% CI = 0.864-0.938); Hosmer-Lemeshow test, *P* = .954; cutoff 5, sensitivity = 91.6% and specificity = 72.7%; cutoff 11, sensitivity = 84.1% and specificity = 86.6%
MDP tool (LASSO model: λ = 0.0085; intercept = −3.13)	History of delirium (coefficient, 0.31); WBC count >12 × 10^9^/L (coefficient, 0.24); creatinine level >1.5 mg/dL (coefficient, 0.18); dementia (coefficient, 0.79); psychiatric disorder (coefficient, 0.13); fall risk score (coefficient, 1.41); ICU admission (coefficient, 1.46)	High, ≥30% predicted probability; moderate, 6%-29% predicted probability; low, ≤5% predicted probability	AUROC, 0.82 (95% CI, 0.81-0.83); Brier score, 0.09; cutoff 30%, sensitivity = 45% and specificity = 93%

Abbreviations: AUROC, area under the receiver operating characteristics curve; ED, emergency department; EMS, emergency medical services; ESI, emergency severity index; ICH, intracranial hemorrhage; ICU, intensive care unit; LASSO, least absolute shrinkage and selection operator; TIA, transient ischemic attack; WBC, white blood count.

SI conversion factor: To convert creatinine to µmol/L, multiply by 88.4.

1. The Kennedy delirium risk prediction rule classifies patients in 3 risk categories: low (≤2 points), moderate (3-4 points) and high (≥5 points).^33^
2. The Zucchelli risk prediction tool, developed using data from patients in the ED observation unit, classifies patients at high and low risk for delirium proposing 2 different cutoffs: 3 points for higher sensitivity and 5 points for higher specificity.^34^
3. The MDP tool was developed for medical inpatients 50 years and older.^35^ We used the same coefficients for the least absolute shrinkage and selection operator (LASSO) model as the original investigators.^28^ This tool classifies patients in 3 categories based on the predicted probability of delirium: low (≤5%), moderate (6%-29%) and high (≥30%).
4. The Recognizing Delirium in Emergency Medicine (REDEEM) score, previously developed in our ED and now reevaluated in a different cohort of patients, classifies patients at high and low risk for delirium proposing 2 different cutoffs: 5 points for higher sensitivity or 11 points for higher specificity.^21^

### Data Collection

All model input variables were electronically extracted from structured data in the EHR. Detailed extracted variables are reported in eMethods 1 in Supplement 1. Race and ethnicity data were included to further investigate the performance of the 4 models across different races and ethnicities and to evaluate the models' generalizability across different populations. Race (White or Other, including American Indian or Alaska Native, Asian, Black or African American, Native Hawaiian or Other Pacific Islander, Other, multiracial, unknown, chose not to disclose, or unable to provide) and ethnicity (Hispanic or non-Hispanic) data were collected as binary variablew because the population was predominantly White and non-Hispanic, and the small sample size of other racial groups prevented us from using more granular demographic groups. Selected codes from *International Classification of Diseases, Ninth Revision,* and *International and Statistical Classification of Diseases, Tenth Revision,* for medical history and selected psychotropic drug use are reported in eTables 1 and 2 in Supplement 1.

Missing data are reported in eTable 3 in Supplement 1. Missing data for blood pressure, respiratory rate, oxygen levels, white blood cell count, and creatinine levels were imputed from the median of the nonmissing data. Emergency severity index codes were imputed as the most frequent code. The missing data for fall risk score, altered elimination, and confusion were also more likely to be negative if not reported in the records and were imputed to be negative or 0. Home medication data were missing either due to the absence of a specific medication among patients’ home medications or true missingness. Home medications were imputed using a random forest single-imputation method. Results not considering missing data and using different imputation methods are shown in eTables 4 to 6 in Supplement 1. For all the imputations, we included patients with no or incomplete DTS and/or bCAM who were then excluded from the analysis.

### Statistical Analysis

Data were analyzed from February 10 to August 21, 2025. Statistical analysis was conducted using R, version 4.5.0 (R Foundation for Statistical Computing).^36^ Descriptive analysis was used to characterize the study population. The Kolmogorov-Smirnov test was used to assess the normality of the continuous data distribution. Continuous data were summarized using the median and IQR. Categorical data were summarized as counts and percentages by category.

For the primary outcome, the AUROC, sensitivity, specificity, positive predictive value (PPV), negative predictive value (NPV), positive likelihood ratio (LR), negative LR, and accuracy, along with their 95% CIs for each model, were computed. Predictive accuracies across demographic subgroups (sex [male or female], race [White or other], and ethnicity [Hispanic or non-Hispanic]) were evaluated for each model. Interval LRs were computed from each score from the 2 × 3 tables.^37^

The McNemar test was used to assess the significance of differences in sensitivities and specificities across the different tools, with a 2-sided *P*< .05 indicating statistical significance.^38^ Furthermore, calibration for each score was assessed. Platt scaling was applied to the Kennedy rule and the Zucchelli tool using logistic regression to derive predictive probabilities from the scoring model.^39^ The method was implemented on a training set (80% of the dataset), and predictive probabilities were subsequently generated for the test set (remaining 20%). Predicted probabilities were derived from coefficients and intercepts for the REDEEM score and MDP tool. The Brier score and Brier skill score were computed for each tool, with BS assessing overall prediction model performance and BSS quantifying improvement over a reference forecast (naive Brier) calculated as BSS = 1 − BS/naïve Brier, where naive Brier assumes the probability of delirium corresponds to the delirium rate in our cohort. Higher BSS values, approaching 1, indicate better model performance. Finally, the Spiegelhalter *z* test was applied to further isolate calibration, with *P* < .05 indicating poor calibration.^39,40,41^

## Results

**Figure 1.**
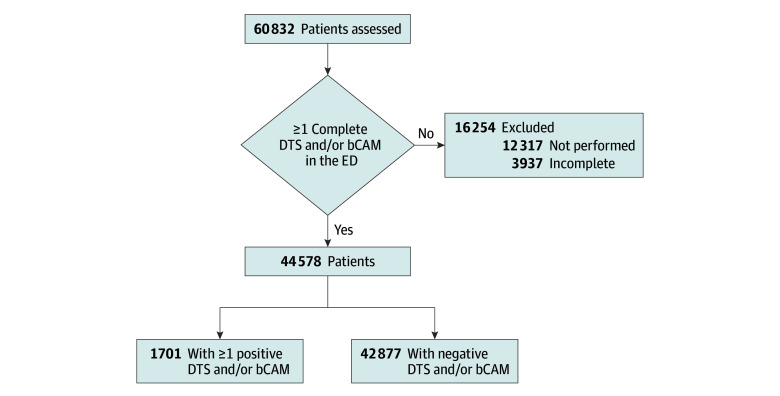
Summary of Patient Population bCAM indicates brief Confusion Assessment Method; DTS, Delirium Triage Screening; and ED, emergency department.

We assessed 37 903 visits from patients 75 years and older between 2021 and 2023 and 22 929 visits from patients 65 years and older in 2024. A total of 12 317 visits (20.2%) were not assessed for delirium, while 3937 (6.5%) had incomplete delirium diagnostic assessments. The final cohort included 44 578 patients, with 1701 (3.8%) diagnosed with delirium (Figure 1). The median age was 82.0 (IQR, 77.5-86.5) years in 2021 to 2023 and 76.0 (IQR, 70.0-82.0) years in 2024. The median age of the entire cohort was 80.0 (IQR, 75.0-85.0) years; 22 786 patients (51.1%) were female, and 21 792 (48.9%) were male. Of the 44 578 patients, 27 075 (60.7%) reported taking a psychotropic drug at home. A description of the study cohort stratified by delirium status is provided in eTable 3 in Supplement 1.

**Figure 2.**
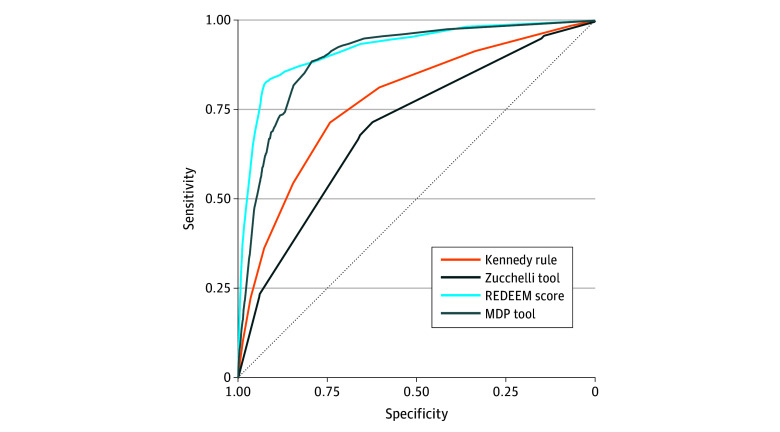
Receiver Operating Characteristics Curves MDP indicates Mayo Delirium Prediction; REDEEM, Recognizing Delirium in Emergency Medicine.

Risk stratification tools were calculated retrospectively for each patient of the whole cohort (N = 44 578). For discrimination, prevalence of delirium using the Kennedy rule included 318 of 26 250 patients (1.2%) in the low-risk group, 455 of 10 790 (4.2%) in the moderate-risk group, and 928 of 7538 (12.3%) in the high-risk group. Prevalence using the Zucchelli tool with a cutoff of 3 included 89 of 6654 patients (1.3%) in the low-risk group and 1612 of 37 924 (4.3%) in the high-risk group. Using a cutoff of 5, the prevalence of delirium included 545 of 28 784 patients (1.9%) in the low-risk group and 1156 of 15 794 (7.3%) high-risk group. Using the MDP tool, prevalence of delirium included 74 of 25 548 patients (0.3%) in the low-risk group, 764 of 15 922 (4.8%) in the moderate-risk group, and 863 of 3108 (27.8%) for the high-risk group. Prevalence using the REDEEM score with a cutoff of 5 included 197 of 34 069 patients (0.6%) in the low-risk group and 1504 of 10 509 (14.3%) in the high-risk group. Prevalence using a cutoff of 11 included 287 of 39 568 patients (0.7%) within the low-risk group and 1414 of 5010 (28.2%) within the high-risk group. Discriminative characteristics for each score are summarized in Table 2 and Figure 2.

**Table 2.** Summarized Risk Score Performances for Different Cutoffs

Discrimination	Kennedy delirium risk prediction rule^a^		Zucchelli risk prediction tool^b^		REDEEM score^c^		MDP tool^d^	
	High vs moderate and low risk	High and moderate vs low risk	Cutoff 3	Cutoff 5	Cutoff 5	Cutoff 11	High vs moderate and low risk	High and moderate vs low risk
Accuracy (95% CI)	0.83 (0.83-0.84)	0.61 (0.61-0.62)	0.18 (0.18-0.19)	0.66 (0.65-0.66)	0.79 (0.79-0.80)	0.91 (0.91-0.92)	0.93 (0.93-0.93)	0.61 (0.60-0.61)
Specificity (95% CI)	0.85 (0.84-0.85)	0.60 (0.60-0.61)	0.15 (0.15-0.16)	0.66 (0.65-0.66)	0.79 (0.79-0.79)	0.92 (0.91-0.92)	0.95 (0.95-0.95)	0.59 (0.59-0.60)
Sensitivity (95% CI)	0.55 (0.52-0.57)	0.81 (0.79-0.83)	0.95 (0.94, 0.96)	0.68 (0.66-0.70)	0.88 (0.87-0.90)	0.83 (0.81-0.85)	0.51 (0.48-0.53)	0.96 (0.95-0.97)
NPV (95% CI)	0.98 (0.98-0.98)	0.99 (0.99-0.99)	0.99 (0.98-0.99)	0.98 (0.98-0.98)	0.99 (0.99-0.99)	0.99 (0.99-0.99)	0.98 (0.98-0.98)	1.00 (1.00-1.00)
PPV (95% CI)	0.12 (0.12-0.13)	0.08 (0.07-0.08)	0.04 (0.04-0.04)	0.07 (0.07-0.08)	0.14 (0.14-0.15)	0.28 (0.27-0.29)	0.28 (0.26-0.29)	0.09 (0.08-0.09)
Negative LR (95% CI)	0.54 (0.51-0.57)	0.31 (0.28-0.34)	0.34 (0.28-0.42)	0.49 (0.45-0.52)	0.15 (0.13-0.17)	0.18 (0.17-0.20)	0.52 (0.50-0.55)	0.07 (0.06-0.09)
Positive LR (95% CI)	3.54 (3.37-3.72)	2.06 (2.01-2.11)	1.12 (1.11- 1.13)	1.99 (1.92-2.06)	4.21 (4.11-4.32)	9.91 (9.54-10.29)	9.69 (9.11-10.31)	2.36 (2.32-2.39)

Abbreviations: LR, likelihood ratio; MDP, Mayo Delirium Prediction; NPV, negative predictive value; PPV, positive predictive value; REDEEM, Recognizing Delirium in Emergency Medicine.

^a^ Area under the receiver operating characteristics curve (AUROC), 0.777 (95% CI, 0.766-0.789).

^b^ AUROC, 0.701 (95% CI, 0.686-0.713).

^c^ AUROC, 0.921 (95% CI, 0.914-0.929).

^d^ AUROC, 0.898 (95% CI, 0.891-0.905).

The Kennedy rule yielded an AUROC of 0.777 (95% CI, 0.766-0.789). At a cutoff of 5, sensitivity was 0.55 (95% CI, 0.52-0.57) and specificity was 85% (95% CI, 84%-85%); positive LR, 3.54 (95% CI, 3.37-3.72); negative LR, 0.54 (95% CI, 0.51-0.57); PPV, 0.12 (95% CI, 0.12-0.13); and NPV, 0.98 (95% CI, 0.98-0.98). The Zucchelli tool achieved an AUROC of 0.701 (95% CI, 0.686-0.713). At a cutoff of 5, sensitivity was 0.68 (95% CI, 0.66-0.70) and specificity was 0.66 (95% CI, 0.65-0.66); positive LR, 1.99 (95% CI, 1.92-2.06); negative LR, 0.49 (95% CI, 0.45-0.52); PPV, 0.07 (95% CI, 0.07-0.08); and NPV, 0.98 (95% CI, 0.98-0.98). The MDP tool yielded an AUROC of 0.898 (95% CI, 0.891-0.905). At a 30% cutoff, sensitivity was 0.51 (95% CI, 0.48-0.53) and specificity was 0.95 (95% CI, 0.95-0.95); positive LR, 9.69 (95% CI, 9.11-10.31); negative LR, 0.52 (95% CI, 0.50-0.55); PPV, 0.28 (95% CI, 0.26-0.29); and NPV, 0.98 (95% CI, 0.98-0.98). The REDEEM score demonstrated the highest AUROC at 0.921 (95% CI, 0.914-0.929). At a cutoff of 11, sensitivity was 0.83 (95% CI, 0.81-0.85) and specificity was 0.92 (95% CI, 0.91-0.92); positive LR, 9.91 (95% CI, 9.54-10.29); negative LR, 0.18 (95% CI, 0.17-0.20); PPV, 0.28 (95% CI, 0.27-0.29); and NPV, 0.99 (95% CI, 0.99-0.99).

Predictive accuracy by demographic subgroup is described in eTable 7 in Supplement 1. Interval LRs with the 2 × 3 tables for each score are shown in eTables 8 to 11 in Supplement 1. Significance for the difference in sensitivity and specificity across the score is summarized in eTables 12 and 13 in Supplement 1.

**Figure 3.**
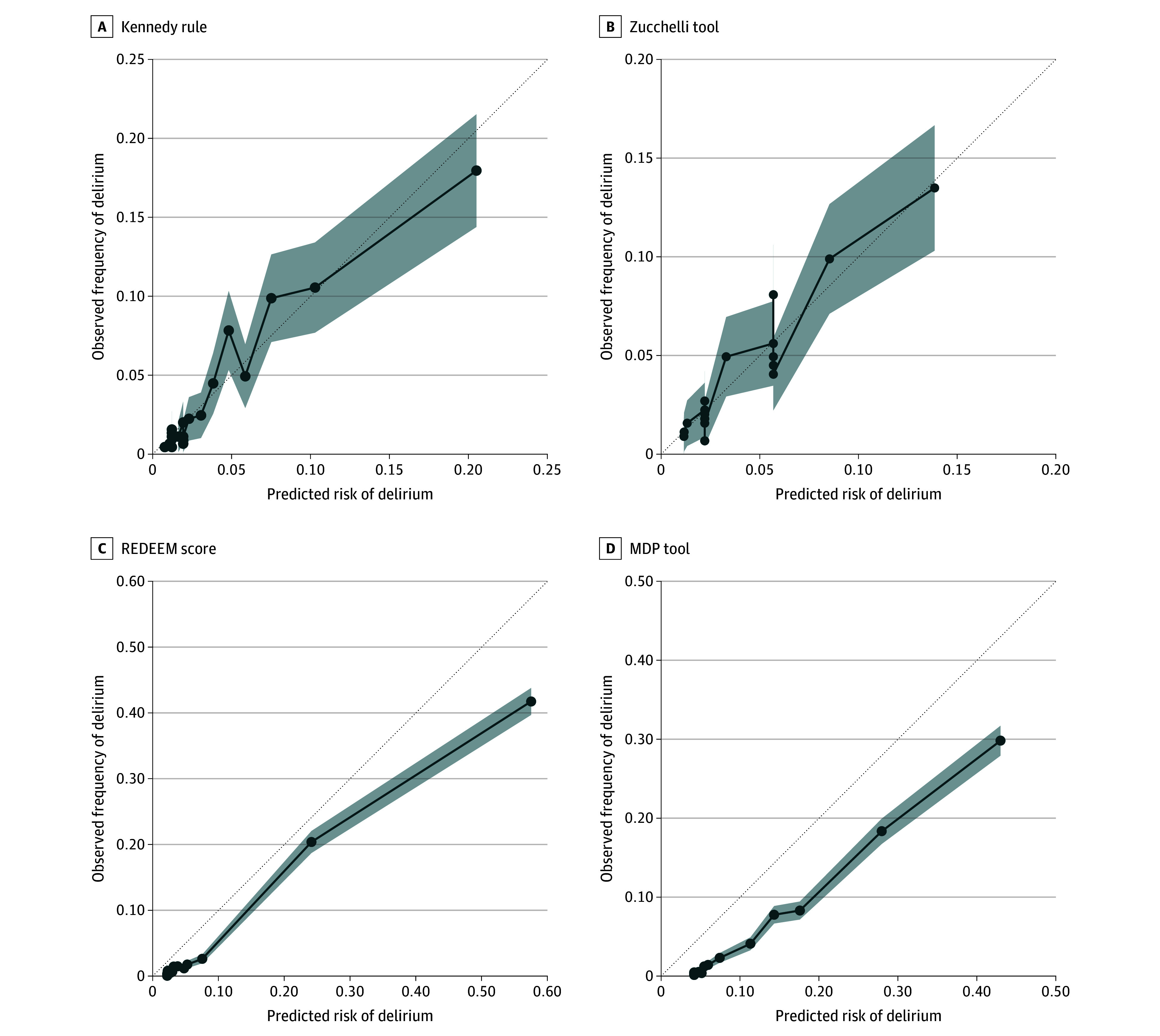
Calibration Plots MDP indicates Mayo Delirium Prediction; REDEEM, Recognizing Delirium in Emergency Medicine.

For calibration, the predicted probability of delirium was derived for the Kennedy rule and Zucchelli tool using Platt scaling. Subsequently, the BS (0.034 for the Kennedy rule and 0.035 for the Zucchelli tool) was applied. For the REDEEM score and MDP tool, the predicted probability was derived from the LASSO model coefficients and intercepts of the original studies (BS, 0.028 for REDEEM and 0.035 for MDP).^21,28^ The results are presented in Figure 3 and eTable 14 in Supplement 1. Using the intercepts from the original studies, the REDEEM score and the MDP tool exhibited slightly higher predicted probabilities compared with the observed frequency of delirium. The REDEEM score and MDP tool were recalibrated using Platt scaling to fit the lower prevalence of delirium in our cohort compared with the original studies.^21,28^ This approach ensures a clearer comparison of the calibration of all 4 tools, as the same methodology was used to derive predicted probabilities for the Kennedy rule and the Zucchelli tool. The recalibration results are presented in eFigure 1 and eTable 15 in Supplement 1.

## Discussion

In this prognostic study, we quantitatively compared the discrimination and calibration of 4 previously developed delirium risk stratification tools. The AUROC in our cohort ranged between 0.701 and 0.921, indicating that each tool exhibited a range of discrimination from potentially helpful (eg, AUROC, 0.600-0.750) to clearly clinically helpful (eg, AUROC >0.750).^42^

Three of the 4 stratification measures demonstrated accuracy consistent with the derivation studies: the Kennedy rule (AUROC, 0.777 vs 0.77 in the original study),^33^ the MDP tool (AUROC, 0.898 vs 0.82 in the validation),^28^ and the REDEEM score (AUROC, 0.921 vs 0.901 in the development study).^21^ The Kennedy rule was externally validated in a previous study using the Delirium Observation Screening Scale, showing a slightly lower AUROC.^43^ The Zucchelli tool showed a lower discrimination (AUROC, 0.701 vs 0.893 in the original study).^34^ This is likely attributable to 2 factors. First, chronic psychotropic drug use was nearly twice as prevalent in our cohort compared with the original test set (60.7% vs 34.6%).^34^ This variation may stem from differences in prescribing practices between the US and Italy. Second, Zucchelli et al^34^ suggest that their model may have been overfitted by selecting variables based on the combination yielding the highest AUROC in the training set, unlike other approaches that used LASSO-penalized logistic regression and univariate feature selection followed by multivariable regression to assign scores based on model coefficients.^21,28,33^ Overall, the REDEEM score demonstrated the strongest predictive capacity, exhibiting the highest AUROC. A cutoff of 11 demonstrated a positive LR of 9.91, thus establishing its potential efficacy in ruling in delirium.^44^ The MDP tool showed high accuracy in predicting delirium with a positive LR comparable with that of the REDEEM score, indicating a similar capability to rule in delirium in these patients. A predicted probability of 5% or less also shows a negative LR of 0.07 being effective to rule out delirium.

Although discrimination is the most reported metric, this measure alone has limited utility to inform selection of the best tool for clinical practice. Calibration is equally important because it reflects how the model predicts the absolute risk of delirium across the spectrum of risk.^24,42,45^ The REDEEM score had the lower BS and the higher BSS, proving to be the best calibrated. Despite this, the calibration curves for the REDEEM score and MDP tool show some overcalling due to the lower prevalence of delirium in our cohort (3.8% vs 11.1%^21^ and 13.8%^28^). The MDP tool has a more comprehensive age cutoff, but hospitalized patients are likely to be more ill, which may explain the different prevalence we found in our cohort. Also, in the original REDEEM study, the screening was performed at the discretion of the bedside nurse, which could have introduced spectrum bias by choosing to screen patients with a higher risk for delirium.^46^ Calibration curves for the Kennedy rule and Zucchelli tool demonstrate robustness, as the predicted probabilities closely correspond to the observed delirium rates. However, this alignment is primarily attributable to Platt scaling, since it would calibrate the probabilities on our dataset. For this reason, we recalibrated the REDEEM score and MDP tool based on our prevalence (eFigure and eTable 15 in Supplement 1).

Overall, the REDEEM score emerged as the best model, using variables generally available at triage (eg, vital signs, reason for ED visit) or usually available within the first hour of stay in the ED (eg, fall risk assessment, history of seizure disorders). Although the MDP tool performed similarly and fits both ED and inpatients settings, it relies on variables not readily available in the ED (eg, laboratory tests, intensive care unit admission). Moreover, the REDEEM score can be integrated within the EHR for automatic scoring using data from the clinical records as they are filled in, enhancing the feasibility of implementation in the ED.

Ideally, EHR tools should provide the exact score or predicted probability, not just high or low risk, to better guide delirium testing and retesting based on sensitivity and specificity thresholds. Because delirium is a fluctuating condition and delirium testing often happens only once and early in the ED stay, an automated system could improve detection rates without requiring dedicated personnel and time.^27,47^

This study represents a step in the clinical implementation of a risk stratification system to improve delirium detection and identify high-risk patients, although effective prevention and treatment strategies await development.^48^ Future research is needed to confirm our results in a more diverse population with multicenter studies and examine the patient-oriented outcomes of ED delirium risk stratification and subsequent interventions targeting delirium treatment or prevention, such as effects on hospitalization, mortality, and duration of delirium.

### Limitations

This study has several limitations. First, the study was conducted in a single academic ED with a predominantly non-Hispanic White patient population; therefore, our findings may not be generalizable to hospitals with different patient populations.^49^ Second, despite efforts to align with the original design, variable abstraction methods differ across studies, potentially affecting consistency. Additionally, to automate psychotropic drug extraction, we curated a drug list that may differ from that used by Zucchelli et al.^34^ Third, we used the DTS and bCAM as a gold standard to determine the presence of delirium. Although the DTS and bCAM are easily applicable by nonspecialists and validated for their use in the ED, they are not the gold standard for delirium identification (represented by *Diagnostic and Statistical Manual of Mental Disorders* [Fifth Edition] criteria), may not identify subtle forms of inattention (key feature for delirium identification), and are limited by the patient or care partner’s preferred language and health literacy.^3,32,49^ Furthermore, the Zucchelli tool and Kennedy rule used different criterion standards for the diagnosis of delirium (4 A’s Test and CAM) that have different detection rates.^3,33,34,50^ Fourth, this study is retrospective, and we used data extracted from the EHR that are less standardized and may be inaccurate or missing.^30,51^ Fifth, the studies by Kennedy et al^33^ and Zucchelli et al^34^ did not include predicted probabilities, and for this reason we applied Platt scaling on the scores to obtain an estimate, but this resulted in a calibration based on our cohort, not allowing comparison of the original calibration with the one of the other tools.

## Conclusions

This prognostic study demonstrated that 3 of 4 existing delirium risk stratification tools—the REDEEM score, MDP tool, and Kennedy rule—exhibited good external validity in an independent ED population, with the REDEEM score and MDP tool achieving the highest discriminative and calibration performance. These findings underscore the importance of external validation and recalibration before clinical implementation, particularly given differences in population characteristics and delirium prevalence. Importantly, tools such as the MDP and the REDEEM score, which are fully automatable within the EHR, offer a feasible and scalable approach to support targeted delirium screening in the ED. These results lay the groundwork for integrating validated risk stratification into ED workflows to improve early delirium detection and inform prevention strategies for high-risk older adults.

## Notes

Supplement 1. **eMethods.** Variables Extracted From the Electronic Health Records (EHR) **eTable 1.** Medical History Diagnosis Codes **eTable 2.** Selected Psychotropic Drugs **eTable 3.** Cohort Description Stratified by Delirium Status With Missing Data **eTable 4.** Results Excluding Patients With Any Missing Data Required for the Tool **eTable 5.** Results Imputing Missing Continuous Variables as Their Median, and Missing Categorical Variables as Negative or 0 **eTable 6.** Results Imputing All Missing Data With a Random Forest Model **eTable 7.** Delirium Prediction Tool Performance Across Patient Demographic Subgroups **eTable 8.** Interval LRs for Kennedy Risk Prediction Rule **eTable 9.** Interval LRs for Zucchelli Risk Prediction Tool **eTable 10.** Interval LRs for REDEEM **eTable 11.** Interval LRs for MDP Tool **eTable 12.** McNemar Test for Differences in Specificities **eTable 13.** McNemar Test for Differences in Sensitivities **eTable 14.** Calibration Characteristics of the 4 Scores **eFigure.** Calibration Plots for REDEEM and MDP Tool After Recalibration **eTable 15.** Calibration Characteristics of REDEEM and MDP Tool After Recalibration With Platt Scaling

Supplement 2.
Data Sharing Statement
