# Supplementary material for: Validation of 4 Risk Stratification Tools for Delirium in the Emergency Department
Source: JAMA Netw Open. 2025 Nov 3;8(11):e2540920. doi: 10.1001/jamanetworkopen.2025.40920 (PMC12584036; doi:10.1001/jamanetworkopen.2025.40920)
Supplement: Supplement 1. — eMethods. Variables Extracted From the Electronic Health Records (EHR) eTable 1. Medical History Diagnosis Codes eTable 2. Selected Psychotropic Drugs eTable 3. Cohort Description Stratified by Delirium Status With Missing Data eTable 4. Results Excluding Patients With Any Missing Data Required for the Tool eTable 5. Results Imputing Missing Continuous Variables as Their Median, and Missing Categorical Variables as Negative or 0 eTable 6. Results Imputing All Missing Data With a Random Forest Model eTable 7. Delirium Prediction Tool Performance Across Patient Demographic Subgroups eTable 8. Interval LRs for Kennedy Risk Prediction Rule eTable 9. Interval LRs for Zucchelli Risk Prediction Tool eTable 10. Interval LRs for REDEEM eTable 11. Interval LRs for MDP Tool eTable 12. McNemar Test for Differences in Specificities eTable 13. McNemar Test for Differences in Sensitivities eTable 14. Calibration Characteristics of the 4 Scores eFigure. Calibration Plots for REDEEM and MDP Tool After Recalibration eTable 15. Calibration Characteristics of REDEEM and MDP Tool After Recalibration With Platt Scaling [file jamanetwopen-e2540920-s001.pdf]

Supplementary Online Content

Bartolacci M, Carpenter KP, Jeffery MM, Mullan AF, Carpenter CR, Bellolio F. Validation of 4 risk stratification tools for delirium in the emergency department. *JAMA Netw Open.* 2025;8(11):e2540920. doi:10.1001/jamanetworkopen.2025.40920

eMethods. Variables Extracted From the Electronic Health Records (EHR)

eTable 1. Medical History Diagnosis Codes

eTable 2. Selected Psychotropic Drugs

eTable 3. Cohort Description Stratified by Delirium Status With Missing Data

eTable 4. Results Excluding Patients With Any Missing Data Required for the Tool

eTable 5. Results Imputing Missing Continuous Variables as Their Median, and Missing Categorical Variables as Negative or 0

eTable 6. Results Imputing All Missing Data With a Random Forest Model

eTable 7. Delirium Prediction Tool Performance Across Patient Demographic Subgroups

eTable 8. Interval LRs for Kennedy Risk Prediction Rule

eTable 9. Interval LRs for Zucchelli Risk Prediction Tool

eTable 10. Interval LRs for REDEEM

eTable 11. Interval LRs for MDP Tool

eTable 12. McNemar Test for Differences in Specificities

eTable 13. McNemar Test for Differences in Sensitivities

eTable 14. Calibration Characteristics of the 4 Scores

eFigure. Calibration Plots for REDEEM and MDP Tool After Recalibration

eTable 15. Calibration Characteristics of REDEEM and MDP Tool After Recalibration With Platt Scaling

This supplementary material has been provided by the authors to give readers additional information about their work.

© 2025 Bartalcci M et al. *JAMA Network Open*.

eMethods. Variables extracted from the Electronic Health Records (EHR)

- Age: in years at the time of the ED visit
- Means of arrival to ED: ambulance or other
- ESI code assigned by the nurse during triage
- History of delirium, dementia, psychiatric disorder, seizures, ischemic stroke or TIA and hearing impairment: ICD9 and ICD10 codes as listed in **eTable1**

eTable 1. Medical History Diagnosis Codes

**Past medical history**	**Diagnosis codes**
**Delirium[a]**	**ICD-10:**<br>F05,F01.51,F02.81,F03.91,F10.221,F10.231,F10.921,F10.96,F10.121,F11.121,F11.221,F11.921,F12.121,F12.221,F12.921,F13.121,F13.221,F13.231,F13.921,F13.931,F14.121,F14.221,F14.921,F15.121,F15.221,F15.921,F16.121,F16.221,F16.921,F18.121,F18.221,F18.921,F19.121,F19.221,F19.231,F19.921,F19.931,R41.0,F43.0<br>**ICD-9:**<br>290.11,290.3,290.41,291.0,291.1,292.81,292.89,293.0,293.1, 293.89,293.9,308.9,780.09
**Dementia[a]**	**ICD-10:**<br>F01.50,F01.51,F02.80,F02.81,F03.90,F03.91,F05,G30.0,G30.1,G30.8,G30.9,G31.01,G31.09,G31.1,G31.83<br>**ICD-9:**<br>290,290.0,290.1,290.10,290.11,290.12,290.13,290.2,290.20,290.21,290.3,290.4,290.40,290.41,290.42,290.43,290.8,290.9,294.1,294.10,294.11,294.20,294.21,294.8,294.9,331,331.0,331.1,331.11,331.19,331.2,331.3,331.4,331.5,331.6,331.7,331.8,331.81,331.82,331.83,331.89,331.9
**Psychiatric disorder[a]: (Psychosis, Schizophrenia, & Depression/Anxiety)**	**ICD-10:**<br>F23,F24,F25.0,F25.1,F25.8,F25.9,F28,F29,F20.0,F20.1,F20.2,F20.3,F20.5,F20.81,F20.89,F20.9,F84.5,F06.31,F06.32,F25.0,F25.1,F30.10,F30.11,F30.12,F30.13,F30.2,F30.3,F30.4,F30.8,F30.9,F31.0,F31.10,F31.11,F31.12,F31.13,F31.2,F31.30,F31.31,F31.32,F31.4,F31.5,F31.60,F31.61,F31.62,F31.63,F31.64,F31.70,F31.71,F31.72,F31.73,F31.74,F31.75,F31.76,F31.77,F31.78,F31.81,F31.89,F31.9,F32.0,F32.1,F32.2,F32.3,F32.4,F32.5,F32.8,F32.81,F32.89,F32.9,F33.0,F33.1,F33.2,F33.3,F33.40,F33.41,F33.42,F33.8,F33.9,F34.0,F34.1,F34.8,F34.81,F34.89,F34.9,F39,F41.8,F43.21,F43.23,F51.13,F53,O90.6,Z86.59<br>**ICD-9:**<br>290,290.0,290.1,290.10,290.11,290.12,290.13,290.2,290.20,290.21,290.3,290.4,290.40,290.41,290.42,290.43,290.8,290.9,291,291.0,291.1,291.2,291.3,291.4,291.5,291.8,291.81,291.82,291.89,291.9,292,292.0,292.1,292.11,292.12,292.2,292.8,292.81,292.82,292.83,292.84,292.85,292.89,292.9,293,293.0,293.1,293.8,293.81,293.82,293.83,293.84,293.89,293.9,294,294.0,294.1,294.10,294.11,294.20,294.21,294.8,294.9,295,295.0,295.00,295.01,295.02,295.03,295.04,295.05,295.1,295.10,295.11,295.12,295.13,295.14,295.15,295.2,295.20,295.21,295.22,295.23,295.24,295.25,295.3,295.30,295.31,295.32,295.33,295.34,295.35,295.4,295.40,295.41,295.42

© 2025 Bartalcci M et al. *JAMA Network Open*.

eTable 1 (continued)

Past medical history	Diagnosis codes
**Psychiatric disorder[a]: (Psychosis, Schizophrenia, & Depression/Anxiety)**	**ICD-9:**<br><br>290,290.0,290.1,290.10,290.11,290.12,290.13,290.2,290.20,290.21,290.3,290.4,290.40,290.41,290.42,290.43,290.8,290.9,291,291.0,291.1,291.2,291.3,291.4,291.5,291.8,291.81,291.82,291.89,291.9,292,292.0,292.1,292.11,292.12,292.2,292.8,292.81,292.82,292.83,292.84,292.85,292.89,292.9,293,293.0,293.1,293.8,293.81,293.82,293.83,293.84,293.89,293.9,294,294.0,294.1,294.10,294.11,294.20,294.21,294.8,294.9,295,295.0,295.00,295.01,295.02,295.03,295.04,295.05,295.1,295.10,295.11,295.12,295.13,295.14,295.15,295.2,295.20,295.21,295.22,295.23,295.24,295.25,295.3,295.30,295.31,295.32,295.33,295.34,295.35,295.4,295.40,295.41,295.42,295.43,295.44,295.45,295.5,295.50,295.51,295.52,295.53,295.54,295.55,295.6,295.60,295.61,295.62,295.63,295.64,295.65,295.7,295.70,295.71,295.72,295.73,295.74,295.75,295.8,295.80,295.81,295.82,295.83,295.84,295.85,295.9,295.90,295.91,295.92,295.93,295.94,295.95,296,296.0,296.00,296.01,296.02,296.03,296.04,296.05,296.06,296.1,296.10,296.11,296.12,296.13,296.14,296.15,296.16,296.2,296.20,296.21,296.22,296.23,296.24,296.25,296.26,296.3,296.30,296.31,296.32,296.33,296.34,296.35,296.36,296.4,296.40,296.41,296.42,296.43,296.44,296.45,296.46,296.5,296.50,296.51,296.52,296.53,296.54,296.55,296.56,296.6,296.60,296.61,296.62,296.63,296.64,296.65,296.66,296.7,296.8,296.80,296.81,296.82,296.89,296.9,296.90,296.99,297,297.0,297.1,297.2,297.3,297.8,297.9,298,298.0,298.1,298.2,298.3,298.4,298.8,298.9,299,299.0,299.00,299.01,299.1,299.10,299.11,299.8,299.80,299.81,299.9,299.90,299.91,648.4,648.40,648.41,648.42,648.43,648.44<br><br>295,295.0,295.00,295.01,295.02,295.03,295.04,295.05,295.1,295.10,295.11,295.12,295.13,295.14,295.15,295.2,295.20,295.21,295.22,295.23,295.24,295.25,295.3,295.30,295.31,295.32,295.33,295.34,295.35,295.4,295.40,295.41,295.42,295.43,296.26,296.3,296.30,296.31,296.32,296.33,296.34,296.35,296.36,296.4,296.40,296.41,296.42,296.43,296.44,296.45,296.46,296.5,296.50,296.51,296.52,296.53,296.54,296.55,296.56,296.6,296.60,296.61,296.62,296.63,296.64,296.65,296.66,296.7,296.8,296.80,296.81,296.82,296.89,296.9,296.90,296.99,298.0,300.11,300.4,301.1,301.10,301.11,301.12,301.13,306.8,308.0,309.0,309.1,309.4,311,313.1,648.42,648.44,V79.0
**History of Seizure Disorders (Epilepsy)[b]**	**ICD-10 / CPT4 / HCPCS Codes**<br>DX G40.001, G40.009, G40.011, G40.019, G40.101, G40.109, G40.111, G40.119, G40.201, G40.209, G40.211, G40.219, G40.301, G40.309, G40.311, G40.319, G40.A01, G40.A09, G40.A11, G40.A19, G40.B01, G40.B09, G40.B11, G40.B19, G40.401, G40.409, G40.411, G40.419, G40.42, G40.501, G40.509, G40.801, G40.802, G40.803, G40.804, G40.811, G40.812, G40.813, G40.814, G40.821, G40.822, G40.823, G40.824, G40.833, G40.834, G40.89, G40.901, G40.909, G40.911, G40.919 (any DX on the claim)<br><br>**ICD-9 / CPT4 / HCPCS Codes**<br>DX 345, 345.0, 345.00, 345.01, 345.1, 345.10, 345.11, 345.2, 345.3, 345.4, 345.40, 345.41, 345.5, 345.50, 345.51, 345.6, 345.60, 345.61, 345.7, 345.70, 345.71, 345.8, 345.80, 345.81, 345.9, 345.90, 345.91 (any DX on the claim)
**History of stroke or TIA[b]**	**ICD-10 / CPT4 / HCPCS Codes**<br>DX G45.0, G45.1, G45.2, G45.8, G45.9, G46.0, G46.1, G46.2, G46.3, G46.4, G46.5, G46.6, G46.7, G46.8, G97.31, G97.32, I60.00, I60.01, I60.02, I60.10, I60.11, I60.12, I60.20, I60.21, I60.22, I60.30, I60.31, I60.32, I60.4, I60.50, I60.51, I60.52, I60.6,

© 2025 Bartalcci M et al. *JAMA Network Open*.

eTable 1 (continued)

Past medical history	Diagnosis codes
**History of stroke or TIA**[b]	**ICD-10 / CPT4 / HCPCS Codes**<br>I60.7, I60.8, I60.9, I61.0, I61.1, I61.2, I61.3, I61.4, I61.5, I61.6, I61.8, I61.9, I63.00, I63.02, I63.011, I63.012, I63.013, I63.019, I63.02, I63.031, I63.032, I63.039, I63.09, I63.10, I63.111, I63.112, I63.119, I63.12, I63.131, I63.132, I63.139, I63.19, I63.20, I63.211, I63.212, I63.213, I63.219, I63.22, I63.231, I63.232, I63.233, I63.239, I63.29, I63.30, I63.311, I63.312, I63.313, I63.319, I63.321, I63.322, I63.323, I63.329, I63.331, I63.332, I63.333, I63.339, I63.341, I63.342, I63.343, I63.349, I63.39, I63.40, I63.411, I63.412, I63.413, I63.419, I63.421, I63.422, I63.423, I63.429, I63.431, I63.432, I63.433, I63.439, I63.441, I63.442, I63.443, I63.449, I63.49, I63.50, I63.511, I63.512, I63.513, I63.519, I63.521, I63.522, I63.523, I63.529, I63.531, I63.532, I63.533, I63.539, I63.541, I63.542, I63.543, I63.549, I63.59, I63.6, I63.8, I63.9, I66.01, I66.02, I66.03, I66.09, I66.11, I66.12, I66.13, I66.19, I66.21, I66.22, I66.23, I66.29, I66.3, I66.8, I66.9, I67.841, I67.848, I67.89, I97.810, I97.811, I97.820, I97.821 (any DX on the claim)<br><br>EXCLUSION: If any of the qualifying claims have any of the following codes in any DX position then EXCLUDE: S01.90XA, S02.0XXA, S02.0XXB, S02.10XA, S02.10XB, S02.101A, S02.101B, S02.102A, S02.102B, S02.109A, S02.109B, S02.11GA, S02.11GB, S02.11HA, S02.11HB, S02.110A, S02.111A, S02.112A, S02.113A, S02.110B, S02.111B, S02.112B, S02.113B, S02.118A, S02.118B, S02.119A, S02.119B, S02.121A, S02.121B, S02.121D, S02.121G, S02.121K, S02.121S, S02.122A, S02.122B, S02.122D, S02.122G, S02.122K, S02.122S, S02.129A, S02.129B, S02.129D, S02.129G, S02.129K, S02.129S, S02.19XA, S02.19XB, S02.2XXA, S02.2XXB, S02.3XXA, S02.30XA, S02.3XXB, S02.30XB, S02.31XA, S02.31XB, S02.32XA, S02.32XB, S02.40AA, S02.40AB, S02.40BA, S02.40BB, S02.40CA, S02.40CB, S02.40DA, S02.40DB, S02.40EA, S02.40EB, S02.40FA, S02.40FB, S02.400A, S02.400B, S02.401A, S02.401B, S02.402A, S02.402B, S02.411A, S02.411B, S02.412A, S02.412B, S02.413A, S02.413B, S02.42XA, S02.42XB, S02.600A, S02.600B, S02.601A, S02.601B, S02.602A, S02.602B, S02.609A, S02.609B, S02.61XA, S02.610A, S02.610B, S02.611A, S02.611B, S02.612A, S02.612B, S02.62XA, S02.620A, S02.62XB, S02.620B, S02.621A, S02.621B, S02.622A, S02.622B, S02.63XA, S02.630A, S02.63XB, S02.630B, S02.631A, S02.631B, S02.632A, S02.632B, S02.64XA, S02.640A, S02.64XB, S02.640B, S02.641A, S02.641B, S02.642A, S02.642B, S02.65XA, S02.650A, S02.65XB, S02.650B, S02.651A, S02.651B, S02.652A, S02.652B, S02.66XA, S02.66XB, S02.67XA, S02.670A, S02.670B, S02.671A, S02.671B, S02.672A, S02.672B, S02.69XA, S02.61XB, S02.62XA, S02.63XA, S02.64XA, S02.65XA, S02.66XA, S02.67XB, S02.69XB, S02.8XXA, S02.80XA, S02.8XXB, S02.80XB, S02.81XA, S02.81XB, S02.82XA, S02.82XB, S02.831A, S02.831B, S02.831D, S02.831G, S02.831K, S02.831S, S02.832A, S02.832B, S02.832D, S02.832G, S02.832K, S02.832S, S02.839A, S02.839B, S02.839D, S02.839G, S02.839K, S02.839S, S02.841A, S02.841B, S02.841D, S02.841G, S02.841K, S02.841S, S02.842A, S02.842B, S02.842D, S02.842G, S02.842K, S02.842S, S02.849A, S02.849B, S02.849D, S02.849G, S02.849K, S02.849S, S02.85XA, S02.85XB, S02.85XD, S02.85XG, S02.85XK, S02.85XS, S02.91XA, S02.91XB, S02.92XA, S02.92XB, S06.0X0A, S06.0X1A, S06.0X2A, S06.0X3A, S06.0X4A, S06.0X5A, S06.0X6A, S06.0X7A, S06.0X8A, S06.0X9A, S06.1X0A, S06.1X1A, S06.1X2A, S06.1X3A, S06.1X4A, S06.1X5A, S06.1X6A, S06.1X7A, S06.1X8A, S06.1X9A, S06.2X0A, S06.2X1A, S06.2X2A, S06.2X3A, S06.2X4A, S06.2X5A, S06.2X6A, S06.2X7A, S06.2X8A, S06.2X9A, S06.2X0B, S06.2X1B, S06.2X2B, S06.2X3B,

© 2025 Bartalcci M et al. *JAMA Network Open*.

eTable 1 (continued)

Past medical history	Diagnosis codes
**History of stroke or TIA**[b]	EXCLUSION: If any of the qualifying claims have any of the following codes in any DX position then EXCLUDE: S06.2X4B, S06.2X5B, S06.2X6B, S06.2X7B, S06.2X8B, S06.2X9B, S06.300A, S06.301A, S06.302A, S06.303A, S06.304A, S06.305A, S06.306A, S06.307A, S06.308A, S06.309A, S06.310A, S06.311A, S06.312A, S06.313A, S06.314A, S06.315A, S06.316A, S06.317A, S06.318A, S06.319A, S06.320A, S06.321A, S06.322A, S06.323A, S06.324A, S06.325A, S06.326A, S06.327A, S06.328A, S06.329A, S06.330A, S06.331A, S06.332A, S06.333A, S06.334A, S06.335A, S06.336A, S06.337A, S06.338A, S06.339A, S06.340A, S06.341A, S06.342A, S06.343A, S06.344A, S06.345A, S06.346A, S06.347A, S06.348A, S06.349A, S06.350A, S06.351A, S06.352A, S06.353A, S06.354A, S06.355A, S06.356A, S06.357A, S06.358A, S06.359A, S06.360A, S06.361A, S06.362A, S06.363A, S06.364A, S06.365A, S06.366A, S06.367A, S06.368A, S06.369A, S06.370A, S06.371A, S06.372A, S06.373A, S06.374A, S06.375A, S06.376A, S06.377A, S06.378A, S06.379A, S06.380A, S06.381A, S06.382A, S06.383A, S06.384A, S06.385A, S06.386A, S06.387A, S06.388A, S06.389A, S06.4X0A, S06.4X1A, S06.4X2A, S06.4X3A, S06.4X4A, S06.4X5A, S06.4X6A, S06.4X7A, S06.4X8A, S06.4X9A, S06.5X0A, S06.5X1A, S06.5X2A, S06.5X3A, S06.5X4A, S06.5X5A, S06.5X6A, S06.5X7A, S06.5X8A, S06.5X9A, S06.6X0A, S06.6X1A, S06.6X2A, S06.6X3A, S06.6X4A, S06.6X5A, S06.6X6A, S06.6X7A, S06.6X8A, S06.6X9A, S06.810A, S06.811A, S06.812A, S06.813A, S06.814A, S06.815A, S06.816A, S06.817A, S06.818A, S06.819A, S06.820A, S06.821A, S06.822A, S06.823A, S06.824A, S06.825A, S06.826A, S06.827A, S06.828A, S06.829A, S06.890A, S06.891A, S06.892A, S06.893A, S06.894A, S06.895A, S06.896A, S06.897A, S06.898A, S06.899A, S06.9X0A, S06.9X1A, S06.9X2A, S06.9X3A, S06.9X4A, S06.9X5A, S06.9X6A, S06.9X7A, S06.9X8A, S06.9X9A, OR Z51.89 as the principal DX Code then EXCLUDE.<br><br>**ICD-9 / CPT4 / HCPCS Codes**<br>DX 430, 431, 433.01, 433.11, 433.21, 433.31, 433.81, 433.91, 434.00, 434.01, 434.10, 434.11, 434.90, 434.91, 435.0, 435.1, 435.3, 435.8, 435.9, 436, 997.02 (any DX on the claim) EXCLUSION: If any of the qualifying claims have: 800 <= DX Code <= 804.9, 850 <= DX Code <= 854.1 in any DX position OR DX V57xx as the principal DX Code, then EXCLUDE.
**Sensory – Deafness and Hearing Impairment**[b]	**ICD-10 / CPT4 / HCPCS Codes**<br>DX H90.3, H90.41, H90.42, H90.5, H90.6, H90.71, H90.72, H90.8, H90.A21, H90.A22, H90.A31, H90.A32, H91.01, H91.02, H91.03, H91.09, H91.3, H91.8X1, H91.8X2, H91.8X3, H91.8X9, H91.90, H91.91, H91.92, H91.93 (any DX on the claim)<br><br>**ICD-9 / CPT4 / HCPCS Codes**<br>DX 389, 389.1, 389.10, 389.11, 389.12, 389.13, 389.14, 389.15, 389.16, 389.17, 389.18, 389.2, 389.20, 389.21, 389.22, 389.7, 389.8, 389.9 (any DX on the claim)

[a]As in Pagali et al., 2022[1]

[b]List of ICD-9 and ICD-10 codes as defined in the Chronic Conditions Data Warehouse of the Center for Medicare and Medicaid Services (CMS).[2,3]

- Chronic use of psychotropic drugs: history of treatment with medications listed **eTable2**

eTable 2. Selected Psychotropic Drugs

DRUG CLASS[a]	INCLUDED MEDICATIONS
**Antiepileptic drugs**	Levetiracetam, Butalbital, Phenobarbital, Primidone, Gabapentin, Pregabalin[b]<br>Brivaracetam, carbamazepine, eslicarbazepine, ethosuximide, felbamate, lacosamide, lamotrigine, methsuximide, oxcarbazepine, perampanel, phenytoin, fosphenytoin, primidone, rufinamide, tiagabine, topiramate, valproic acid/ divalproex, vigabatrin, zonisamide[c]
**Hypnotics**	Alprazolam, Chlordiazepoxide, Clobazam, Clonazepam, Clorazepate, Diazepam, Estazolam, Lorazepam, Midazolam, Oxazepam, Temazepam, Triazolam, Eszopiclone, Zaleplon, Zolpidem, Trazodone[b]<br>Buspirone, Chloral hydrate, Lemborexant, Meprobamate, Oxybate, Suvorexant, Diphenhydramine, Doxylamine, Hydroxyzine[d]
**Antipsychotics**	Chlorpromazine, Fluphenazine, Haloperidol, Perphenazine<br>Aripiprazole, Brexpiprazole, Cariprazine, Clozapine, Lurasidone, Olanzapine, Paliperidone, Pimavanserin, Quetiapine, Risperidone, Ziprasidone[b]
**Antidepressants (SNRI, SSRI, atypical, tricyclics)**	Amitriptyline, Amoxapine, Clomipramine, Desipramine, Doxepin, Imipramine, Nortriptyline, Paroxetine, Mirtazapine[b]<br>Trimipramine, Protriptyline, Maprotiline, Sertraline, Fluvoxamine, Fluoxetine, Paroxetine, Citalopram, Escitalopram, Venlafaxine, Desvenlafaxine, Duloxetine, Milnacipran, Levomilnacipran, Bupropion, Agomelatine[e]
**Anti-dementia drugs**	Ergoloid mesylates, Donepezil, Galantamine, Rivastigmine[b]<br>Lecanemab, Donanemab
**Opioids**	Tramadol<br>Oxycodone, hydrocodone, morphine, codeine, fentanyl[f]
**Parkinson's medications**	Benztropine, Trihexyphenidyl[b]<br>Carbidopa-levodopa, Foscarbidopa/ foslevodopa, Levodopa, Entacapone, Tolcapone, Opicapone, Pramipexole, Ropinirole, Apomorphine, Rotigotine, Selegiline, Rasagiline, Safinamide, Amantadine, Istradefylline[g]

[a]As in Zucchelli et al.,2021[4]
[b]List of drugs from the BEER criteria[5]
[c]List from Vossler D. G et al.,2018[6]
[d]List from Sedatives and Hypnotics - LiverTox - NCBI Bookshelf [Updated 2018 Feb 20][7]
[e]List from Sheffler ZM et al.[8]
[f]List from NIH National Institute of Drug Abuse[9]
[g]List from the American Parkinson Association[10]

- Fall risk assessment: performed by ED nurses with the MEDFRAT Fall Risk Assessment Tool showed below.[11] We considered the result as binary: high risk or not high risk.

© 2025 Bartalcci M et al. *JAMA Network Open*.

History of falling in the last 3 months, including since admission	0 points (No)<br>1 point (Yes, single mechanical fall)<br>2 points (Yes, physiological fall/syncope/dizziness)<br>3 points (Yes, fall prone – multiple falls)
Is the patient confused or disoriented?	0 points (No)<br>5 points (Yes)
Is the patient intoxicated or sedated?	0 points (No)<br>3 points (Yes)
Does the patient have an impaired gait?	0 points (No)<br>1 point (Yes)
Does the patient use a mobility assistance device?	0 points (No)<br>1 point (Yes)
Is the patient experiencing altered elimination?	0 points (No)<br>1 point (Yes)
Total score	0 to 14<br>Low risk: 0 to 2 points<br>High risk: ≥ 3 points

- Vital signs: first listed in the EMR
- WBC count: LOINC CODE 6690-2[1]
- Creatinine: LOINC CODE 14682-9, 2160, 45066-8, 38483-4, 35203-9, 59826-8, 59834-2[1]
- Suspected infection in the ED: ED diagnosis or patient chief complaint
- ED diagnosis of intracranial hemorrhage (ICH): ICD-10 codes I61-I62 from the ED diagnosis

eTable 3. Cohort Description Stratified by Delirium Status With Missing Data

	**Total sample**				**2021-2023**				**2024**			
**Variable**	**Patients with delirium** (N=1701)	**Patients without delirium** (N=42887)	**Total** (N= 44578)	**Missing values**, n (%)	**Patients with delirium** (N=1197)	**Patients without delirium** (N=25885)	**Total** (N=27082)	**Missing values**, n (%)	**Patients with delirium** (N=504)	**Patients without delirium** (N=16992)	**Total** (N=17496)	**Missing values**, n (%)
**Age**, median (Q1-Q3)	82 (76.5-87.5)	80 (75.5-84.5)	80 (75-85)	0 (0%)	83 (78-88)	82 (77.5-86.5)	82 (77.5-86.5)	0 (0%)	80 (74-86)	75 (69-81)	76 (70-82)	0 (0%)
**Sex,** n females (%)	913 (53.67%)	21873 (51.0%)	22786 (51.11%)	0 (0%)	651 (54.39%)	13380 (51.69%)	14031 (82.14%)	0 (0%)	405 (80.36%)	8350 (49.14%)	8755 (50.04%)	0 (0%)
**Triage information**												
**EMS arrival**, n (%)	1240 (72.9%)	15871 (37.02%)	17111 (38.38%)	0 (0%)	879 (73.43%)	10519 (40.64%)	11398 (42.09%)	0 (0%)	361 (71.63%)	5352 (31.5%)	5713 (32.65%)	0 (0%)
**Chief complaint of altered mental status**, n (%)	638 (37.51%)	855 (1.99%)	1493 (3.35%)	0 (0%)	441 (36.84%)	571 (2.21%)	1012 (3.74%)	0 (0%)	197 (39.09%)	284 (1.67%)	481 (2.75%)	0 (0%)
**Triage ESI**, n (%)				12 (0.03%)				8 (0.03%)				4 (0.02%)
Level 1 (most severe)	13 (0.76%)	60 (0.14%)	73 (0.16%)		8 (0.67%)	46 (0.18%)	54 (0.20%)		5 (0.99%)	14 (0.08%)	19 (0.11%)	
Level 2	551 (32.39%)	6436 (15.01%)	6987 (15.67%)		359 (30.00%)	3902 (15.07%)	4261 (15.73%)		192 (38.10%)	2534 (14.91%)	2726 (15.58%)	

eTable 3 (continued)

© 2025 Bartalcci M et al. *JAMA Network Open*.

	Total sample				2021-2023				2024			
**Variable**	**Patients with delirium** (N=1701)	**Patients without delirium** (N=42887)	**Total** (N= 44578)	**Missing values**, n (%)	**Patients with delirium** (N=1197)	**Patients without delirium** (N=25885)	**Total** (N=27082)	**Missing values**, n (%)	**Patients with delirium** (N=504)	**Patients without delirium** (N=16992)	**Total** (N=17496)	**Missing values**, n (%)
Level 3	1118 (65.73%)	32560 (75.92%)	33678 (75.44%)		815 (68.09%)	19570 (75.60%)	20385 (75.27%)		303 (60.12%)	12990 (76.45%)	13293 (75.98%)	
Level 4	18 (1.06%)	3777 (8.81%)	3795 (8.51%)		15 (1.25%)	2339 (9.04%)	2354 (8.69%)		3 (0.60%)	1438 (8.46%)	1441 (8.24%)	
Level 5 (least severe)	0 (0%)	33 (0.08%)	33 (0.07%)		0 (0%)	20 (0.08%)	20 (0.07%)		0 (0%)	13 (0.08%)	13 (0.07%)	
**Vital signs**												
**First systolic blood pressure**, median (Q1-Q3)	139 (120-158)	142 (125-159)	142 (124.5-159.5)	52 (0.12%)	139 (120-158)	142 (125-159)	142 (125-159)	33 (0.07%)	138 (120-156)	141 (124-158)	141 (124-158)	19 (0.04%)
**First diastolic blood pressure**, median (Q1-Q3)	76 (64.37-87.6)	76 (66-86)	76 (65.5-86.5)	52 (0.12%)	76 (64.5-87.5)	75 (64.5-85.5)	75 (64.5-85.5)	33 (0.07%)	78 (66-90)	77 (67-87)	77 (67-87)	19 (0.04%)
**First respiratory rate**, median (Q1-Q3)	18 (15-21)	18 (16-20)	18 (16-20)	1577 (3.54%)	18 (15-21)	18 (16-20)	18 (16-20)	919 (2.06%)	18 (15-21)	18 (16-20)	18 (16-20)	658 (1.48%)

eTable 3 (continued)

© 2025 Bartalcci M et al. *JAMA Network Open*.

	Total sample				2021-2023				2024			
**Variable**	**Patients with delirium** (N=1701)	**Patients without delirium** (N=42887)	**Total** (N= 44578)	**Missing values,** n (%)	**Patients with delirium** (N=1197)	**Patients without delirium** (N=25885)	**Total** (N=27082)	**Missing values,** n (%)	**Patients with delirium** (N=504)	**Patients without delirium** (N=16992)	**Total** (N=17496)	**Missing values,** n (%)
**First oxygen saturation (%)**, median (Q1-Q3)	96 (94-98)	97 (95.5-98.5)	97 (95.5-98.5)	82 (0.18%)	96 (94-98)	97 (95.5-98.5)	97 (95.5-98.5)	44 (0.1%)	96 (94-98)	97 (95.5-98.5)	97 (95.5-98.5)	38 (0.09%)
**Labs**												
**White blood count**, median (Q1-Q3)	8.6 (6.1-11.2)	8 (5.9-10.1)	8 (5.9-10.1)	6660 (14.94%)	8.6 (6.1-11.1)	8 (5.6-10.1)	8 (5.9-10.1)	4043 (9.07%)	8.8 (6.1-11.5)	7.9 (5.8-10)	7.9 (5.8-10)	2617 (5.87%)
**Creatinine**, median (Q1-Q3)	1.07 (0.77-1.38)	1.01 (0.75-1.27)	1.02 (0.76-1.28)	6778 (15.2%)	1.07 (0.77-1.37)	1.03 (0.77-1.30)	1.03 (0.76-1.3)	4124 (9.25%)	1.08 (0.44-1.4)	0.99 (0.75-1.24)	1 (0.75-1.25)	2654 (5.95%)
**Fall risk score**												
**High fall risk** n (%)	1292 (82.39 %)	4840 (11.53%)	6132 (14.09 %)	1045 (2.34%)	928 (83.23 %)	3569 (14.12%)	4497 (17.04%)	694 (1.56%)	364 (80%)	1271 (0.08%)	1635 (0.1%)	351 (0.79%)
**Altered elimination,** n (%)	783 (49.65 %)	5819 (13.85%)	6602 (15.15 %)	988 (2.22%)	573 (51.21 %)	3979 (15.72%)	4552 (17.22%)	654 (1.47%)	210 (45.85 %)	1840 (11.02%)	2050 (11.94%)	334 (0.75%)
**Confusion**, n (%)	1261 (80.01 %)	2563 (6.1%)	3824 (8.77%)	981(2.2 %)	908 (81.22 %)	2007 (7.93%)	2915 (11.03%)	649 (1.46%)	353 (77.07 %)	556 (3.33%)	909 (5.3%)	332 (0.74%)
**Past medical history**												
**Delirium**, n (%)	1093 (64.26 %)	7942 (18.52%)	9035 (20.27 %)	0 (0%)	785 (65.58 %)	5369 (20.74%)	6154 (22.72%)	0 (0%)	308 (61.11%)	2573 (15.14%)	2881 (16.47%)	0 (0%)

eTable 3 (continued)

© 2025 Bartalcci M et al. *JAMA Network Open*.

	Total sample				2021-2023				2024			
**Variable**	**Patients with delirium** (N=1701)	**Patients without delirium** (N=42887)	**Total** (N= 44578)	**Missing values,** n (%)	**Patients with delirium** (N=1197)	**Patients without delirium** (N=25885)	**Total** (N=27082)	**Missing values,** n (%)	**Patients with delirium** (N=504)	**Patients without delirium** (N=16992)	**Total** (N=17496)	**Missing values,** n (%)
**Dementia**, n (%)	1038 (61.02 %)	6618 (15.43%)	7656 (17.17 %)	0 (0%)	745 (62.24 %)	4768 (18.42%)	5513 (20.36%)	0 (0%)	293 (58.13 %)	1850 (10.89%)	2143 (12.25%)	0 (0%)
**Psychiatric disorder**, n (%)	701 (41.21 %)	12481 (29.11%)	13182 (29.57 %)	0 (0%)	493 (41.19 %)	7551 (29.17%)	8044 (29.7%)	0 (0%)	208 (41.27 %)	4930 (29.01%)	5138 (29.37%)	0 (0%)
**Seizure**, n (%)	181 (10.64 %)	1514 (3.53%)	1695 (3.8%)	0 (0%)	117 (9.77%)	986 (3.81%)	1103 (4.07%)	0 (0%)	64 (12.7%)	528 (3.11%)	592 (3.38%)	0 (0%)
**Hearing Impairment**, n (%)	588 (34.57 %)	12257 (28.59%)	12845 (28.81 %)	0 (0%)	435 (36.34 %)	8162 (31.53%)	8597 (31.74%)	0 (0%)	153 (30.36 %)	4095 (24.1%)	4248 (24.28%)	0 (0%)
**Ischemic stroke or TIA**, n (%)	255 (14.99 %)	1882 (4.39%)	2137 (4.79%)	0 (0%)	199 (16.62 %)	1278 (4.94%)	1477 (5.45%)	0 (0%)	56 (11.11%)	604 (3.55%)	660 (3.77%)	0 (0%)
**ED diagnosis**												
**ED diagnosis of ICH**, n (%)	8 (0.47%)	53 (0.12%)	61 (0.14%)		6 (0.5%)	42 (0.16%)	48 (0.18%)	0 (0%)	2 (0.4%)	11 (0.06%)	13 (0.07%)	0 (0%)
**ED suspected infection**, n (%)	180 (10.58 %)	2966 (6.92%)	3146 (7.06%)	0 (0%)	135 (11.28 %)	1943 (7.51%)	2078 (7.67%)	0 (0%)	45 (8.93%)	1023 (6.02%)	1068 (6.1%)	0 (0%)
**Home medication**												
**Psychotropic drugs**, n (%)	1232 (72.42 %)	25843 (60.26%)	27075 (60.74 %)	17503 (39.36 %)	867 (72.43 %)	15639 (60.42%)	16506 (60.95%)	10576 (39.05 %)	365 (72.42 %)	10204 (60.05%)	10569 (60.41%)	6927 (39.59 %)

eTable 3 (continued)

© 2025 Bartalcci M et al. *JAMA Network Open*.

	Total sample				2021-2023				2024			
**Variable**	**Patients with delirium** (N=1701)	**Patients without delirium** (N=42887)	**Total** (N= 44578)	**Missing values,** n (%)	**Patients with delirium** (N=1197)	**Patients without delirium** (N=25885)	**Total** (N=27082)	**Missing values,** n (%)	**Patients with delirium** (N=504)	**Patients without delirium** (N=16992)	**Total** (N=17496)	**Missing values,** n (%)
**ED disposition**												
**Admit to ICU**, n (%)	223 (13.11%)	1403 (3.27%)	1626 (3.65%)	0 (0%)	154 (12.87%)	902 (3.48%)	1056 (3.9%)	0 (0%)	69 (13.69%)	501 (2.95%)	570 (3.26%)	0 (0%)
**Abbreviations: EMS=Emergency Medical Services; TIA=Transient Ischemic Attack ICH = Intracerebral Hemorrhage; ICU= Instensive Care Unit**												

© 2025 Bartalcci M et al. *JAMA Network Open*.

eTable 4. Results Excluding Patients With Any Missing Data Required for the Tool

	**Kennedy's Delirium Risk Prediction Rule 1655/43001**		**Zucchelli's Risk Prediction Tool 1232/27075**		**REDEEM 1527/41954**		**MDP TOOL 1492/36561**	
**AUC-ROC (CI95%)**	0.778 (0.766-0.789)		0.696 (0.682-0.711)		0.931 (0.924-0.939)		0.902 (0.895-0.909)	
	High risk **VS** moderate and low risk	High and moderate risk **VS** low risk	Cutoff 3	Cutoff 5	Cutoff 5	Cutoff 11	High risk VS Moderate and low risk	High and moderate risk VS low risk
**ACCURACY (CI95%)**	0.83 (0.83-0.84)	0.61 (0.61-0.62)	0.17 (0.17-0.18)	0.62 (0.62-0.63)	0.79 (0.79-0.80)	0.91 (0.91-0.92)	0.93 (0.92-0.93)	0.58 (0.57-0.58)
**SPECIFICITY (CI95%)**	0.85 (0.84-0.85)	0.60 (0.60-0.61)	0.14 (0.13-0.14)	0.62 (0.61-0.62)	0.79 (0.79-0.79)	0.92 (0.91-0.92)	0.94 (0.94-0.95)	0.56 (0.56-0.57)
**SENSITIVITY (CI95%)**	0.55 (0.52-0.57)	0.81 (0.79-0.83)	0.96 (0.95, 0.97)	0.71 (0.68-0.73)	0.91 (0.89-0.92)	0.86 (0.84-0.88)	0.54 (0.51-0.57)	0.96 (0.95-0.97)
**NPV (CI95%)**	0.98 (0.98,0.98)	0.99 (0.99-0.99)	0.99 (0.98-0.99)	0.98 (0.98-0.98)	1.00 (0.99-1.00)	0.99 (0.99-1.00)	0.98 (0.98-0.98)	1.00 (1.00-1.00)
**PPV (CI95%)**	0.12 (0.12-0.13)	0.08 (0.07-0.08)	0.05 (0.05-0.05)	0.08 (0.08-0.09)	0.14 (0.13-0.15)	0.28 (0.26-0.29)	0.29 (0.27-0.30)	0.09 (0.08-0.09)
**LR- (CI95%)**	0.54 (0.51-0.56)	0.31 (0.28-0.34)	0.28 (0.21-0.37)	0.47 (0.43-0.52)	0.12 (0.10-0.14)	0.15 (0.13-0.17)	0.49 (0.46-0.52)	0.07 (0.05-0.09)
**LR+ (CI95%)**	3.55 (3.38-3.73)	2.05 (2.00-2.11)	1.11 (1.10, 1.13)	1.85 (1.78-1.92)	4.30 (4.19-4.41)	10.17 (9.79-10.56)	9.42 (8.85-10.04)	2.20 (2.17-2.24)

eTable 5. Results Imputing Missing Continuous Variables as Their Median, and Missing Categorical Variables as Negative or 0

© 2025 Bartalcci M et al. *JAMA Network Open*.

	Kennedy's Delirium Risk Prediction Rule		Zucchelli's Risk Prediction Tool		REDEEM		MDP TOOL	
**AUC-ROC (CI95%)**	0.777 (0.766-0.789)		0.713 (0.700-0.725)		0.921 (0.914-0.929)		0.898 (0.891-0.905)	
	High risk **VS** moderate and low risk	High and moderate risk **VS** low risk	Cutoff 3	Cutoff 5	Cutoff 5	Cutoff 11	High risk VS Moderate and low risk	High and moderate risk VS low risk
**ACCURACY (CI95%)**	0.83 (0.83-0.84)	0.61 (0.61-0.62)	0.38 (0.37-0.38)	0.66 (0.65-0.66)	0.79 (0.79-0.80)	0.91 (0.91-0.92)	0.93 (0.93-0.93)	0.61 (0.60-0.61)
**SPECIFICITY (CI95%)**	0.85 (0.84-0.85)	0.60 (0.60-0.61)	0.36 (0.35-0.36)	0.66 (0.65-0.66)	0.79 (0.79-0.79)	0.92 (0.91-0.92)	0.95 (0.95-0.95)	0.59 (0.59, 0.60)
**SENSITIVITY (CI95%)**	0.55 (0.52-0.57)	0.81 (0.79-0.83)	0.87 (0.85, 0.88)	0.68 (0.66-0.70)	0.88 (0.87-0.90)	0.83 (0.81-0.85)	0.51 (0.48-0.53)	0.96 (0.95, 0.97)
**NPV (CI95%)**	0.98 (0.98,0.98)	0.99 (0.99-0.99)	0.99 (0.98-0.99)	0.98 (0.98-0.98)	0.99 (0.99-0.99)	0.99 (0.99-0.99)	0.98 (0.98-0.98)	1.00 (1.00, 1.00)
**PPV (CI95%)**	0.12 (0.12-0.13)	0.08 (0.07-0.08)	0.05 (0.05-0.05)	0.07 (0.07-0.08)	0.14 (0.14-0.15)	0.28 (0.27-0.29)	0.28 (0.26-0.29)	0.09 (0.08, 0.09)
**LR- (CI95%)**	0.54 (0.51-0.57)	0.31 (0.28-0.34)	0.37 (0.32-0.41)	0.49 (0.45-0.52)	0.15 (0.13-0.17)	0.18 (0.17-0.20)	0.52 (0.50-0.55)	0.07 (0.06, 0.09)
**LR+ (CI95%)**	3.54 (3.37-3.72)	2.06 (2.01-2.11)	1.35 (1.33-1.38)	1.99 (1.92-2.06)	4.21 (4.11-4.32)	9.91 (9.54-10.29)	9.69 (9.11-10.31)	2.36 (2.32, 2.39)

eTable 6. Results Imputing All Missing Data With a Random Forest Model

	Kennedy's Delirium Risk Prediction Rule	Zucchelli's Risk Prediction Tool	REDEEM	MDP TOOL

© 2025 Bartalcci M et al. *JAMA Network Open*.

**AUC-ROC (CI95%)**	0.777 (0.765-0.788)		0.702 (0.690-0.714)		0.926 (0.919-0.933)		0.903 (0.896-0.909)	
	High risk **VS** moderate and low risk	High and moderate risk **VS** low risk	Cutoff 3	Cutoff 5	Cutoff 5	Cutoff 11	High risk VS Moderate and low risk	High and moderate risk VS low risk
**ACCURACY (CI95%)**	0.83 (0.83-0.84)	0.61 (0.61-0.62)	0.17 (1.17-0.18)	0.62 (0.62-0.63)	0.79 (0.79-0.80)	0.91 (0.91-0.92)	0.93 (0.92-0.93)	0.93 (0.92-0.93)
**SPECIFICITY (CI95%)**	0.84 (0.84-0.85)	0.60 (0.60-0.61)	0.15 (0.15-0.16)	0.66 (0.65-0.66)	0.79 (0.78-0.79)	0.91 (0.91-0.92)	0.95 (0.94-0.95)	0.58 (0.57-0.58)
**SENSITIVITY (CI95%)**	0.55 (0.52-0.57)	0.81 (0.79-0.83)	0.95 (0.94, 0.96)	0.68 (0.66-0.70)	0.89 (0.88-0.91)	0.85 (0.83-0.86)	0.53 (0.51-0.55)	0.96 (0.95-0.97)
**NPV (CI95%)**	0.98 (0.98-0.98)	0.99 (0.98-0.99)	0.99 (0.98-0.99)	0.98 (0.98-0.98)	1.00 (0.99-1.00)	0.99 (0.99-0.99)	0.98 (0.98-0.98)	1.00 (1.00-1.00)
**PPV (CI95%)**	0.12 (0.12-0.13)	0.08 (0.07-0.08)	0.04 (0.04-0.04)	0.07 (0.07-0.08)	0.14 (0.14-0.15)	0.28 (0.27-0.29)	0.28 (0.26-0.29)	0.08 (0.08-0.09)
**LR- (CI95%)**	0.54 (0.51-0.57)	0.31 (0.28-0.34)	0.32 (0.26-0.39)	0.49 (0.45-0.52)	0.13 (0.12-0.15)	0.17 (0.15-0.19)	0.50 (0.47-0.52)	0.07 (0.05-0.08)
**LR+ (CI95%)**	3.52 (3.35-3.69)	2.05 (2.00-2.10)	1.12 (1.11, 1.14)	1.99 (1.92-2.06)	4.23 (4.13-4.34)	9.91 (9.55-10.29)	9.76 (9.19-10.36)	2.27 (2.24-2.30)

© 2025 Bartalcci M et al. *JAMA Network Open*.

Subgroups analysis

eTable 7. Delirium Prediction Tool Performance Across Patient Demographic Subgroups

	Kennedy's Delirium Risk Prediction Rule		Zucchelli's Risk Prediction Tool		REDEEM		MDP Tool	
	High risk VS moderate and low risk	High and moderate risk VS low risk	Cutoff 3	Cutoff 5	Cutoff 5	Cutoff 11	High risk VS moderate and low risk	High and moderate risk VS low risk
**Males** (N = 21,792); **Delirium prevalence 3.6%**								
**AUC-ROC**	0.770 (0.752-0.788)		0.697 (0.679-0.715)		0.914 (0.902-0.926)		0.893 (0.882-0.903)	
**Accuracy**	0.84 (0.84-0.85)	0.62 (0.61-0.63)	0.19 (0.18-0.19)	0.66 (0.66-0.67)	0.79 (0.79-0.80)	0.92 (0.91-0.92)	0.93 (0.93-0.94)	0.59 (0.59-0.60)
**Specificity**	0.85 (0.85-0.86)	0.61 (0.61-0.62)	0.16 (0.16-0.17)	0.66 (0.66-0.67)	0.79 (0.78-0.80)	0.92 (0.92-0.92)	0.95 (0.95-0.95)	0.58 (0.58-0.59)
**Sensitivity**	0.54 (0.50-0.57)	0.80 (0.77-0.82)	0.94 (0.92,0.95)	0.67 (0.64-0.71)	0.88 (0.85-0.90)	0.81 (0.79-0.84)	0.50 (0.46-0.53)	0.96 (0.94-0.97)
**NPV**	0.98 (0.98-0.98)	0.99 (0.99-0.99)	0.99 (0.98,0.99)	0.98 (0.98-0.98)	0.99 (0.99-1.00)	0.99 (0.99-0.99)	0.98 (0.98-0.98)	1.00 (1.00-1.00)
**PPV**	0.12 (0.11-0.13)	0.07 (0.07-0.08)	0.04 (0.04-0.04)	0.07 (0.06-0.08)	0.14 (0.13-0.15)	0.28 (0.26-0.30)	0.27 (0.24-0.29)	0.08 (0.07-0.08)
**LR-**	0.54 (0.50-0.59)	0.33 (0.29-0.38)	0.38 (0.29-0.50)	0.49 (0.45-0.55)	0.15 (0.13-0.19)	0.20 (0.17-0.23)	0.53 (0.49-0.57)	0.08 (0.06-0.11)
**LR+**	3.65 (3.39-3.92)	2.06 (1.98-2.15)	1.12 (1.10-1.14)	1.99 (1.89-2.10)	4.18 (4.03-4.34)	10.18 (9.62-10.77)	9.72 (8.87-10.64)	2.28 (2.23-2.33)
**Females** (N = 22,786); **Delirium prevalence 4.0%**								
**AUC-ROC**	0.783 (0.768-0.798)		0.704 (0.688-0.721)		0.927 (0.918-0.936)		0.902 (0.892-0.911)	
**Accuracy**	0.83 (0.82-0.83)	0.60 (0.60-0.61)	0.18 (0.17-0.18)	0.66 (0.65-0.66)	0.79 (0.79-0.80)	0.91 (0.91-0.91)	0.93 (0.93-0.93)	0.62 (0.61-0.63)
**Specificity**	0.84 (0.83-0.84)	0.60 (0.59-0.60)	0.14 (0.14-0.15)	0.66 (0.65-0.66)	0.79 (0.78-0.80)	0.91 (0.91-0.92)	0.95 (0.94-0.95)	0.61 (0.60-0.61)
**Sensitivity**	0.55 (0.52-0.59)	0.83 (0.80-0.85)	0.96 (0.94-0.97)	0.69 (0.65-0.72)	0.89 (0.87-0.91)	0.85 (0.82-0.87)	0.51 (0.48-0.55)	0.96 (0.94-0.97)
**NPV**	0.98 (0.98-0.98)	0.99 (0.99-0.99)	0.99 (0.98-0.99)	0.98 (0.98-0.98)	0.99 (0.99-1.00)	0.99 (0.99-0.99)	0.98 (0.98-0.98)	1.00 (1.00-1.00)
**PPV**	0.13 (0.12-0.14)	0.08 (0.07-0.08)	0.04 (0.04-0.05)	0.08 (0.07-0.08)	0.15 (0.14-0.16)	0.29 (0.27-0.30)	0.29 (0.27-0.31)	0.09 (0.09-0.10)

© 2025 Bartalcci M et al. *JAMA Network Open*.

eTable 7 (continued)

	Kennedy's Delirium Risk Prediction Rule		Zucchelli's Risk Prediction Tool		REDEEM		MDP Tool	
	High risk VS moderate and low risk	High and moderate risk VS low risk	Cutoff 3	Cutoff 5	Cutoff 5	Cutoff 11	High risk VS moderate and low risk	High and moderate risk VS low risk
**LR-**	0.53 (0.50-0.57)	0.29 (0.25-0.33)	0.30 (0.22-0.41)	0.48 (0.44-0.53)	0.14 (0.12-0.17)	0.17 (0.15-0.20)	0.51 (0.48-0.55)	0.07 (0.05-0.10)
**LR+**	3.44 (3.22-3.67)	2.05 (1.98-2.12)	1.12 (1.10-1.13)	1.99 (1.90-2.09)	4.24 (4.09-4.39)	9.66 (9.18-10.16)	9.65 (8.87-10.50)	2.44 (2.39-2.49)
**White patients** (N = 42,422); **Delirium prevalence 3.8%**								
**AUC-ROC**	0.777 (0.765-0.789)		0.702 (0.689-0.714)		0.922 (0.914-0.929)		0.898 (0.891-0.905)	
**Accuracy**	0.83 (0.83-0.84)	0.61 (0.60-0.61)	0.18 (0.17-0.18)	0.65 (0.65-0.66)	0.79 (0.79-0.80)	0.91 (0.91-0.92)	0.93 (0.93-0.93)	0.61 (0.60-0.61)
**Specificity**	0.84 (0.84-0.85)	0.60 (0.60-0.61)	0.15 (0.14-0.15)	0.65 (0.65-0.66)	0.79 (0.78-0.79)	0.92 (0.91-0.92)	0.95 (0.94-0.95)	0.59 (0.59-0.60)
**Sensitivity**	0.55 (0.52-0.57)	0.82 (0.80-0.83)	0.95 (0.94-0.96)	0.69 (0.66-0.71)	0.89 (0.87-0.90)	0.83 (0.81-0.85)	0.51 (0.49-0.54)	0.96 (0.95-0.97)
**NPV**	0.98 (0.98-0.98)	0.99 (0.99-0.99)	0.99 (0.98-0.99)	0.98 (0.98-0.98)	0.99 (0.99-1.00)	0.99 (0.99-0.99)	0.98 (0.98-0.98)	1.00 (1.00-1.00)
**PPV**	0.12 (0.11-0.13)	0.07 (0.07-0.08)	0.04 (0.04-0.04)	0.07 (0.07-0.08)	0.14 (0.13-0.15)	0.28 (0.27-0.29)	0.28 (0.26-0.29)	0.08 (0.08-0.09)
**LR-**	0.54 (0.51-0.57)	0.31 (0.28-0.34)	0.33 (0.27-0.41)	0.48 (0.45-0.52)	0.15 (0.13-0.17)	0.18 (0.17-0.21)	0.51 (0.49-0.54)	0.07 (0.06-0.09)
**LR+**	3.49 (3.32-3.67)	2.05 (1.99-2.10)	1.11 (1.10-1.13)	1.98 (1.91-2.05)	4.19 (4.08-4.30)	9.84 (9.47-10.23)	9.63 (9.05-10.26)	2.34 (2.30-2.38)
**Non-white patients** (N = 1,816); **Delirium prevalence 4.5%**								
**AUC-ROC**	0.788 (0.735-0.841)		0.713 (0.660-0.766)		0.908 (0.870-0.945)		0.892 (0.854-0.930)	
**Accuracy**	0.87 (0.85-0.88)	0.68 (0.65-0.70)	0.32 (0.29-0.34)	0.76 (0.74-0.78)	0.82 (0.80-0.83)	0.93 (0.91-0.94)	0.94 (0.93-0.95)	0.67 (0.65-0.69)
**Specificity**	0.88 (0.87-0.90)	0.67 (0.65-0.69)	0.29 (0.27-0.31)	0.77 (0.75-0.79)	0.82 (0.80-0.83)	0.93 (0.92-0.94)	0.96 (0.95-0.97)	0.66 (0.63-0.68)
**Sensitivity**	0.54 (0.42-0.65)	0.76 (0.65-0.84)	0.93 (0.85-0.97)	0.57 (0.46-0.68)	0.84 (0.74-0.91)	0.82 (0.72-0.89)	0.41 (0.31-0.53)	0.94 (0.86-0.98)
**NPV**	0.98 (0.97-0.98)	0.98 (0.97-0.99)	0.99 (0.97-1.00)	0.97 (0.96-0.98)	0.99 (0.98-1.00)	0.99 (0.98-0.99)	0.97 (0.96-0.98)	1.00 (0.99-1.00)
**PPV**	0.18 (0.13-0.23)	0.10 (0.08-0.12)	0.06 (0.05-0.07)	0.11 (0.08-0.14)	0.18 (0.14-0.22)	0.36 (0.29-0.43)	0.35 (0.26-0.46)	0.11 (0.09-0.14)

© 2025 Bartalcci M et al. *JAMA Network Open*.

eTable 7. (continued)

	Kennedy's Delirium Risk Prediction Rule		Zucchelli's Risk Prediction Tool		REDEEM		MDP Tool	
	**High risk** VS **moderate and low risk**	**High and moderate risk** VS **low risk**	**Cutoff 3**	**Cutoff 5**	**Cutoff 5**	**Cutoff 11**	**High risk** VS **moderate and low risk**	**High and moderate risk** VS **low risk**
**LR-**	0.52 (0.41-0.66)	0.36 (0.25-0.53)	0.26 (0.12-0.55)	0.55 (0.43-0.71)	0.19 (0.12-0.32)	0.20 (0.12-0.31)	0.61 (0.51-0.73)	0.09 (0.04-0.22)
**LR+**	4.65 (3.66-5.91)	2.30 (2.00-2.65)	1.30 (1.21-1.39)	2.48 (2.02-3.05)	4.57 (3.99-5.24)	11.71 (9.59-14.30)	11.60 (8.13-16.54)	2.74 (2.51-2.98)
**Non-Hispanic patients** (N = 43,231); **Delirium prevalence 3.8%**								
**AUC-ROC**	0.779 (0.767-0.790)		0.702 (0.690-0.715)		0.921 (0.913-0.928)		0.897 (0.890-0.904)	
**Accuracy**	0.83 (0.83-0.84)	0.61 (0.61-0.62)	0.18 (0.18-0.18)	0.66 (0.65-0.66)	0.79 (0.79-0.80)	0.91 (0.91-0.92)	0.93 (0.93-0.93)	0.61 (0.60-0.61)
**Specificity**	0.84 (0.84-0.85)	0.60 (0.60-0.61)	0.15 (0.15-0.15)	0.66 (0.65-0.66)	0.79 (0.79-0.79)	0.92 (0.91-0.92)	0.95 (0.95-0.95)	0.59 (0.59-0.60)
**Sensitivity**	0.55 (0.53-0.57)	0.82 (0.80-0.83)	0.95 (0.94-0.96)	0.68 (0.66-0.71)	0.88 (0.87-0.90)	0.83 (0.81-0.85)	0.51 (0.48-0.53)	0.96 (0.94-0.96)
**NPV**	0.98 (0.98-0.98)	0.99 (0.99-0.99)	0.99 (0.98-0.99)	0.98 (0.98-0.98)	0.99 (0.99-0.99)	0.99 (0.99-0.99)	0.98 (0.98-0.98)	1.00 (1.00-1.00)
**PPV**	0.12 (0.12-0.13)	0.08 (0.07-0.08)	0.04 (0.04-0.04)	0.07 (0.07-0.08)	0.14 (0.14-0.15)	0.28 (0.27-0.29)	0.28 (0.26-0.29)	0.09 (0.08-0.09)
**LR-**	0.53 (0.51-0.56)	0.31 (0.28-0.34)	0.34 (0.27-0.41)	0.48 (0.45-0.52)	0.15 (0.13-0.17)	0.18 (0.17-0.21)	0.52 (0.49-0.54)	0.08 (0.06-0.09)
**LR+**	3.53 (3.36-3.70)	2.05 (2.00-2.11)	1.12 (1.10-1.13)	1.99 (1.92-2.06)	4.19 (4.09-4.30)	9.88 (9.51-10.27)	9.68 (9.09-10.30)	2.34 (2.31-2.38)
**Hispanic patients** (N = 618); **Delirium prevalence 4.0%**								
**AUC-ROC**	0.637 (0.516-0.758)		0.402 (0.321-0.493)		0.934 (0.866-1.00)		0.926 (0.898-0.954)	
**Accuracy**	0.89 (0.86-0.91)	0.70 (0.66-0.73)	0.24 (0.20-0.27)	0.71 (0.67-0.74)	0.84 (0.81-0.87)	0.95 (0.93-0.96)	0.94 (0.92-0.96)	0.66 (0.62-0.70)
**Specificity**	0.92 (0.89-0.94)	0.70 (0.66-0.74)	0.21 (0.18-0.24)	0.72 (0.68-0.76)	0.84 (0.81-0.87)	0.95 (0.93-0.97)	0.97 (0.95-0.98)	0.64 (0.60-0.68)
**Sensitivity**	0.24 (0.09-0.45)	0.60 (0.39-0.79)	0.92 (0.74-0.99)	0.40 (0.21-0.61)	0.92 (0.74-0.99)	0.88 (0.69-0.97)	0.28 (0.12-0.49)	1.00 (0.86-1.00)
**NPV**	0.97 (0.95-0.98)	0.98 (0.96-0.99)	0.98 (0.94-1.00)	0.97 (0.94-0.98)	1.00 (0.99-1.00)	0.99 (0.98-1.00)	0.97 (0.95-0.98)	1.00 (0.99-1.00)
**PPV**	0.11 (0.04-0.22)	0.08 (0.04-0.12)	0.05 (0.03-0.07)	0.06 (0.03-0.10)	0.20 (0.13-0.28)	0.43 (0.29-0.58)	0.27 (0.12-0.48)	0.11 (0.07-0.15)

© 2025 Bartalcci M et al. *JAMA Network Open*.

eTable 7. (continued)

	Kennedy's Delirium Risk Prediction Rule		Zucchelli's Risk Prediction Tool		REDEEM		MDP Tool	
	High risk VS moderate and low risk	High and moderate risk VS low risk	Cutoff 3	Cutoff 5	Cutoff 5	Cutoff 11	High risk VS moderate and low risk	High and moderate risk VS low risk
**LR-**	0.83 (0.66-1.03)	0.57 (0.35-0.93)	0.38 (0.10-1.46)	0.83 (0.60-1.15)	0.10 (0.03-0.36)	0.13 (0.04-0.36)	0.74 (0.58-0.95)	0.00 (---)[2]
**LR+**	2.90 (1.38-6.13)	2.00 (1.42-2.82)	1.16 (1.03-1.32)	1.43 (0.87-2.35)	5.80 (4.66-7.22)	17.99 (12.26-26.40)	8.74 (4.05-18.85)	2.81 (2.52-3.13)

[1]Patients with unknown demographics were not included in subgroup results
[2]No delirium positive patients were classified as low risk and no CI could be determined

© 2025 Bartalcci M et al. *JAMA Network Open*.

Interval likelihood ratios for each score

eTable 8. Interval LRs for Kennedy Risk Prediction Rule

Kennedy Delirium Risk Prediction Rule	Delirium positives	Delirium Negatives	Total	Interval LR
≥5	928	6610	7538	3.54
3-4	455	10335	10790	1.11
≤2	318	25932	26250	0.31
Total	1701	42877	44578	

eTable 9. Interval LRs for Zucchelli Risk Prediction Tool

Zucchelli Risk Prediction Tool	Delirium positives	Delirium Negatives	Total	Interval LR
≥5	1156	14638	15794	1.99
2-4	456	21674	22130	0.53
≤3	89	6565	6654	0.34
Total	1701	42877	44578	

eTable 10. Interval LRs for REDEEM

REDEEM	Delirium positives	Delirium Negatives	Total	Interval LR
≥11	1414	3596	5010	9.91
6-10	90	5409	5499	0.42
≤5	197	33872	34069	0.15
Total	1701	42877	44578	

eTable 11. Interval LRs for MDP Tool

MDP tool	Delirium positives	Delirium Negatives	Total	Interval LR
≥30%	863	2245	3108	9.69
6-29%	764	15158	15922	1.27
≤5%	74	25474	25548	0.07
Total	1701	42877	44578	

McNemar test for specificities and sensitivities

eTable 12. McNemar Test for Differences in Specificities

**Delirium positives (N=1701)**	**Zucchelli's Risk Prediction Tool (Cutoff 3)**	**Zucchelli's Risk Prediction Tool (Cutoff 5)**	**REDEEM (Cutoff 5)**	**REDEEM (Cutoff 11)**	**MDP tool (Cutoff 30%)**	**MDP tool (Cutoff 5%)**	**Kennedy's Delirium Risk Prediction Rule (Cutoff 5)**	**Kennedy's Delirium Risk Prediction Rule (Cutoff 2)**
**Zucchelli's Risk Prediction Tool (Cutoff 3)**		51%, $p < 2.2e-16$	64%, $p = 1.312e-11$	77%, $p < 2.2e-16$	80%, $p < 2.2e-16$	44%, p= **0.2141**	70%, $p < 2.2e-16$	45%, $p < 2.2e-16$
**Zucchelli's Risk Prediction Tool (Cutoff 5)**			13%, $p < 2.2e-16$	26%, $p < 2.2e-16$	29%, $p < 2.2e-16$	7%, $p < 2.2e-16$	19%, $p < 2.2e-16$	6%, $p < 2.2e-16$
**REDEEM (Cutoff 5)**				13%, $p < 2.2e-16$	16%, $p < 2.2e-16$	20%, $p < 2.2e-16$	6%, $p < 2.2e-16$	19%, $p = 2.066e-09$
**REDEEM (Cutoff 11)**					3%, $p < 2.2e-16$	33%, $p < 2.2e-16$	7%, $p < 2.2e-16$	32%, p =**0.1587**
**MDP tool (cutoff 30%)**						36%, $p < 2.2e-16$	10%, $p = 0.003061$	35%, $p < 2.2e-16$
**MDP tool (cutoff 5%)**							26%, $p < 2.2e-16$	1%, $p < 2.2e-16$
**Kennedy's Delirium Risk Prediction Rule (Cutoff 5)**								25%, $p < 2.2e-16$
**Kennedy's Delirium Risk Prediction Rule (Cutoff 2)**								

eTable 13. McNemar Test for Differences in Sensitivities

© 2025 Bartalcci M et al. *JAMA Network Open*.

Delirium negatives (N=42887)	Zucchelli's Risk Prediction Tool (Cutoff 3)	Zucchelli's Risk Prediction Tool (Cutoff 5)	REDEEM (Cutoff 5)	REDEEM (Cutoff 11)	MDP tool (Cutoff 30%)	MDP tool (Cutoff 5%)	Kennedy's Delirium Risk Prediction Rule (Cutoff 5)	Kennedy's Delirium Risk Prediction Rule (Cutoff 2)
**Zucchelli's Risk Prediction Tool (Cutoff 3)**		27%, p < 2.2e-16	7%, p < 2.2e-16	12%, p < 2.2e-16	44%, p < 2.2e-16	1%, p < 2.2e-16	40%, p < 2.2e-16	14%, p < 2.2e-16
**Zucchelli's Risk Prediction Tool (Cutoff 5)**			20%, p < 2.2e-16	15%, p < 2.2e-16	17%, p < 2.2e-16	28%, p < 2.2e-16	13%, p < 2.2e-16	13%, p < 2.2e-16
**REDEEM (Cutoff 5)**				5%, p < 2.2e-16	37%, p < 2.2e-16	8%, p < 2.2e-16	33%, p < 2.2e-16	7%, p < 2.2e-16
**REDEEM (Cutoff 11)**					32%, p < 2.2e-16	13%, p < 2.2e-16	28%, p < 2.2e-16	2%, p < 2.2e-16
**MDP tool (cutoff 30%)**						45%, p < 2.2e-16	4%, p < 2.2e-16	30%, p < 2.2e-16
**MDP tool (cutoff 5%)**							41%, p < 2.2e-16	15%, p =7.885e-05
**Kennedy's Delirium Risk Prediction Rule (Cutoff 5)**								26%, p < 2.2e-16
**Kennedy's Delirium Risk Prediction Rule (Cutoff 2)**								

Brier score, Brier Skill Score and Spiegelhalter test

eTable 14. Calibration Characteristics of the 4 Scores

© 2025 Bartalcci M et al. *JAMA Network Open*.

**Calibration**				
**BS**	0.034	0.035	0.028	0.035
**BSS**	0.073	0.054	0.237	0.057
**Spiegelhalter z-test**	-0.344	-0.837	-24.842	-42.105
**Spiegelhalter p-value**	0.731	0.402	0	0

Recalibration results of REDEEM and MDP tool

REDEEM and MDP tool were recalibrated using Platt scaling on a training set containing the 80% of the sample. Predicted probabilities were then calculated on a test set containing the remaining 20%. The same method was used to derive predicted probabilities from Zucchelli's and Kennedy's score. Results are shown **eFigure1** and **eTable12** below:

eFigure. Calibration Plots for REDEEM and MDP Tool After Recalibration

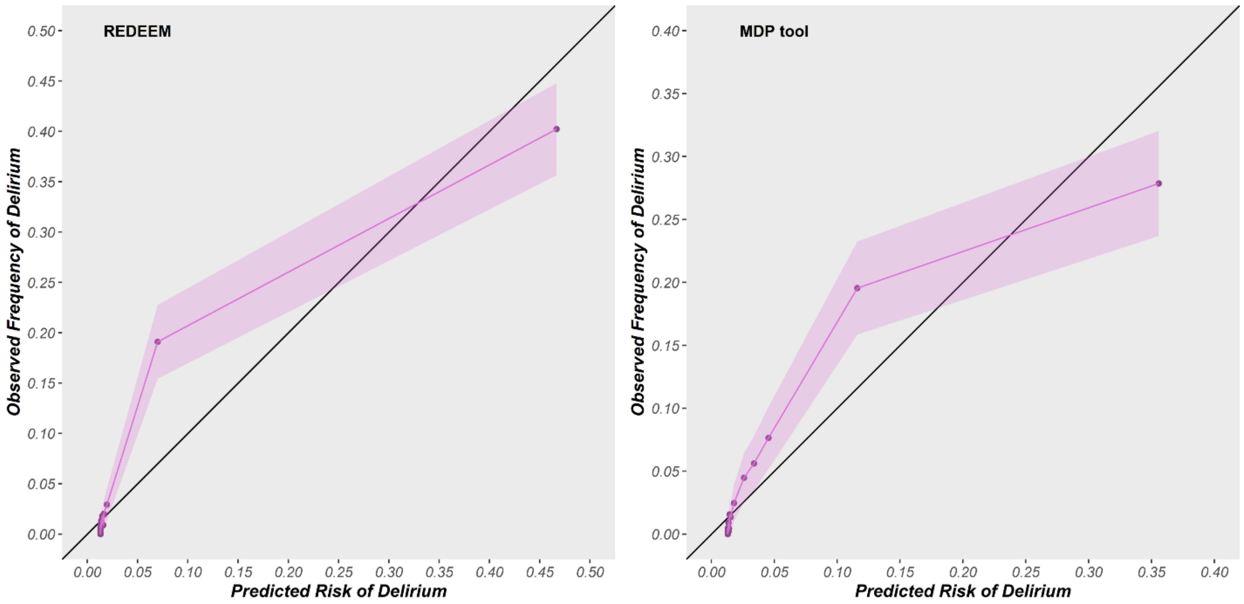

eTable 15. Calibration Characteristics of REDEEM and MDP Tool After Recalibration With Platt Scaling

	Recalibrated REDEEM	Recalibrated MDP tool
**BS**	0.028	0.032
**BSS**	0.237	0.114
**Spiegelhalter z-test**	2.381	2.025
**Spiegelhalter p-value**	0.017	0.043

References

1. Pagali SR, Fischer KM, Kashiwagi DT, et al. Validation and Recalibration of Modified Mayo Delirium Prediction Tool in a Hospitalized Cohort. *J Acad Consult Liaison Psychiatry*. Nov-Dec 2022;63(6):521-528. doi:10.1016/j.jaclp.2022.05.006

2. Centers for Medicare & Medicaid Services. Condition Categories - Chronic Conditions Data Warehouse [Internet]. Accessed February 23, 2021. https://www2.ccwdata.org/web/guest/condition-categories
3. Oliveira JESL, Stanich JA, Jeffery MM, et al. REcognizing DElirium in geriatric Emergency Medicine: The REDEEM risk stratification score. *Acad Emerg Med*. Apr 2022;29(4):476-485. doi:10.1111/acem.14423
4. Zucchelli A, Apuzzo R, Paolillo C, et al. Development and validation of a delirium risk assessment tool in older patients admitted to the Emergency Department Observation Unit. *Aging Clin Exp Res*. Oct 2021;33(10):2753-2758. doi:10.1007/s40520-021-01792-4
5. By the American Geriatrics Society Beers Criteria Update Expert P. American Geriatrics Society 2023 updated AGS Beers Criteria(R) for potentially inappropriate medication use in older adults. *J Am Geriatr Soc*. Jul 2023;71(7):2052-2081. doi:10.1111/jgs.18372
6. Vossler DG, Weingarten M, Gidal BE, American Epilepsy Society Treatments C. Summary of Antiepileptic Drugs Available in the United States of America: WORKING TOWARD A WORLD WITHOUT EPILEPSY. *Epilepsy Curr*. Jul-Aug 2018;18(4 Suppl 1):1-26. doi:10.5698/1535-7597.18.4s1.1
7. LiverTox: Clinical and Research Information on Drug-Induced Liver Injury. Bethesda (MD): National Institute of Diabetes and Digestive and Kidney Diseases; 2012-. Sedatives and Hypnotics. [Internet]. Accessed January 27, 2025. https://www.ncbi.nlm.nih.gov/books/NBK547943/
8. Sheffler ZM PP, Abdijadid S. Antidepressants. [Updated 2023 May 26]. In: StatPearls [Internet]. . Accessed January 27, 2025. https://www.ncbi.nlm.nih.gov/books/NBK538182/
9. NIDA. 2021, June 1. Prescription Opioids DrugFacts. National Institute on Drug Abuse website. Accessed January 27, 2025. https://nida.nih.gov/publications/drugfacts/prescription-opioids
10. Full List of Medications Approved for the Treatment of Parkinson's Disease in the USA [Internet]. Accessed January 27, 2025. https://www.apdaparkinson.org/living-with-parkinsons-disease/treatment-medication/medication/
11. Flarity K, Pate T, Finch H. Development and implementation of the Memorial Emergency Department Fall Risk Assessment Tool. *Adv Emerg Nurs J*. Jan-Mar 2013;35(1):57-66. doi:10.1097/TME.0b013e31827c6a54

© 2025 Bartalcci M et al. *JAMA Network Open*.
