# Supplementary material for: Validation of 4 Risk Stratification Tools for Delirium in the Emergency Department
Source: JAMA Netw Open. 2025 Nov 3;8(11):e2540920. doi: 10.1001/jamanetworkopen.2025.40920 (PMC12584036; doi:10.1001/jamanetworkopen.2025.40920)
Supplement: Supplement 2. — Data Sharing Statement [file jamanetwopen-e2540920-s002.pdf]

Data Sharing Statement

Bartolacci. Validation of 4 Risk Stratification Tools for Delirium in the Emergency Department. *JAMA Netw Open*. Published November 03, 2025. doi:10.1001/jamanetworkopen.2025.40920

Data
Data available: Yes
Data types: Data dictionary, Other (please specify)
Additional Information: Code used for analysis
How to access data: Bellolio.Fernanda@mayo.edu
When available: With publication

Supporting Documents
Document types: Statistical/analytic code
How to access documents: Bellolio.Fernanda@mayo.edu
When available: With publication

Additional Information
Who can access the data: Upon reasonable request and IRB approval
Types of analyses: NA
Mechanisms of data availability: with a signed data access agreement
